# Integrating a Multimodal Digital Device for Continuous Perioperative Monitoring in Patients With Lung Cancer Undergoing Thoracic Surgery: Development and Usability Study

**DOI:** 10.2196/69512

**Published:** 2025-09-16

**Authors:** Runchen Wang, Jianqi Zheng, Wenwei Guo, Haiqi Huang, Qixia Wang, Yihong Li, Manwan Lin, Linchong Huang, Qing Zhang, Kaishen Chen, Zhiming Ye, Hongsheng Deng, Yu Jiang, Yuechun Lin, Yi Feng, Ying Huang, Ying Chen, Jianxing He, Hengrui Liang

**Affiliations:** 1 Department of Thoracic Surgery, China State Key Laboratory of Respiratory Disease & National Clinical Research Center for Respiratory Disease the First Affiliated Hospital of Guangzhou Medical University Guangzhou China; 2 Department of Respiratory and Critical Care Medicine, Guangzhou Institute of Respiratory Health, State Key Laboratory of Respiratory Disease, National Clinical Research Center for Respiratory Disease, National Center for Respiratory Medicine the First Affiliated Hospital of Guangzhou Medical University Guangzhou China; 3 CinoCore AI department Shanghai CinoCore Health Technology Co Ltd Shanghai China

**Keywords:** lung cancer, digital device, wearable device, patients reported outcomes, multi-modal, artificial intelligence, AI

## Abstract

**Background:**

Minimally invasive thoracic surgery has improved lung cancer outcomes but requires enhanced postoperative care. Traditionally, the episodic care model has limited timely and multidimensional monitoring of patients. Recent technological advances in multimodal digital devices, including wearable devices and electronic patient-reported outcomes (ePROs), offer a promising solution to these challenges. However, current studies focus on only a few parameters and limited application in thoracic surgery.

**Objective:**

This study aims to propose a self-controlled study to evaluate the feasibility and reliability of multimodal digital devices, including wearables and ePROs, for continuous perioperative monitoring to enhance recovery after thoracic surgery.

**Methods:**

We included 288 patients with non–small cell lung cancer from the Guangzhou Medical University cohort, which includes 2757 participants with various lung diseases. Digital data were collected during hospitalization using a commercial smartwatch combined with an ePROs questionnaire, while clinical data were obtained from electronic health records (EHRs). Agreement between the digital device and EHR was evaluated via Bland-Altman analysis. Time-series data were normalized for continuous outlier monitoring, and threshold analysis of ePROs scores were used to explore associations across different modules.

**Results:**

Throughout hospitalization, digital devices provided a subjective overview of the patients’ recovery trajectories. Results of Bland-Altman analysis demonstrated a high level of agreement between the digital device and the EHR. For body temperature, the analysis revealed a minimal bias of 0.02 °C (95% CI –0.01 °C to 0.05 °C), the agreement for heart rate showed a bias of 0.26 beats per minute (bpm; 95% CI –0.49 bpm to 1.01 bpm), and the bias for oxygen saturation was –0.06% (95% CI –0.27% to 0.15%), indicating close alignment between the 2 measurement methods. Meanwhile, wearable devices demonstrate significant potential in outlier detection compared to the episodic care model, offering accurate and sensitive monitoring of outliers between traditional measurement intervals. Using a thresholding method, we found that wearable metrics were correlated with the severity of ePROs.

**Conclusions:**

These findings highlight the reliability and clinical potential of digital device–based multimodal systems within the enhanced recovery after surgery framework, offering a novel approach for continuous perioperative monitoring.

## Introduction

### Background

Minimally invasive thoracic surgery (MITS) has significantly improved perioperative outcomes for patients with lung cancer, offering benefits such as reduced postoperative pain, shorter hospital stays, and quicker recovery times [1]. However, the success of MITS also demands higher standards for postoperative care, with a focus on early mobilization, increased physical activity, and personalized rehabilitation [2,3]. Building upon the foundation of smaller incisions, the enhanced recovery after surgery (ERAS) protocol further emphasizes both the speed and quality of recovery [4].

Currently, the commonly used perioperative monitoring model in clinical practice is episodic care, such as bedside monitoring, periodic assessment, and ward rounds. However, this care model limits patients’ activities and timely feedback on subjective feelings. Moreover, some abnormal signs of intermittent attacks may be missed due to the discontinuity of monitoring. Therefore, a novel pattern to obtain multimodal, comprehensive, real-time, continuous, and reliable data of patient condition is urgently needed to achieve ERAS.

Recent technological advances in digital devices, including wearable devices and electronic patient-reported outcomes (ePROs) systems, offer a promising solution to these challenges [5,6]. Wearable devices, such as smartwatches and smart rings, can be used for continuous health monitoring, providing a more comprehensive assessment of an individual’s well-being [7]. ePROs, in turn, can obtain real-time subjective feedback from patients. Previous studies have demonstrated that ePROs can reduce postoperative symptom burden and complications [8-10] and have shown their prognostic and therapeutic benefits [11].

Despite these advances, several challenges still remain. First, most studies focus on the continuous tracking of only a few parameters, predominantly heart rate (HR) and step count. Second, previous research has mainly concentrated on cardiovascular disease, as HR monitoring is relatively straightforward. Finally, there is a scarcity of studies combining wearable devices with ePROs for use in clinical practice, particularly in thoracic surgery.

### Objective

To address the gaps mentioned earlier, we propose a prospective, self-controlled study to explore the feasibility and reliability of using multimodal digital devices, including wearable technology combined with ePROs, and provide a novel evidence-based approach to continuous perioperative monitoring for enhanced recovery after thoracic surgery.

## Methods

### Ethical Considerations

This prospective study received approval from the Ethics Committee of the National Center for Respiratory Medicine and the First Affiliated Hospital of Guangzhou Medical University (ES-2024-073-02) and was registered with the clinical trial registry (NCT06118229). All data incorporated in this study were deidentified. As compensation, participants in this study received regular follow-up visits and one-on-one counselling services provided by the medical team upon completion of the study. This study followed the Declaration of Helsinki.

### Patients Selection

All patients were drawn from the Guangzhou Medical University cohort, a prospective, multicenter cohort designed to use multimodal data, including intelligent digital data, biospecimen data, and imaging artificial intelligence data from 2757 participants with various lung diseases, to assist in diagnosis, monitor treatment efficacy, and promote the rapid recovery of surgical patients. In this study, we prospectively and consecutively recruited patients with non–small cell lung cancer, one of the study arms within the Guangzhou Medical University cohort, aged ≥18 years, who were scheduled to undergo thoracic surgery between March 2023 and December 2023.

Detailed exclusion criteria were as follows: (1) patients unable to wear the wearable device or have poor compliance; (2) patients with a previous history of trauma, infection, or tumor; (3) patients without a smartphone equipped with an Android (Google LLC) or iOS (Apple Inc) operating system; (4) patients with other systemic inflammatory or neurological diseases; and (5) pregnant or lactating women.

All eligible patients provided informed consent after receiving detailed written information about the study, including the purpose of the study and use of wearable devices. Both verbal and written informed consent was obtained.

### Wearable Device

The HUAWEI WATCH D (Huawei Device Co, Ltd) is a wrist-worn class II medical device approved by the Chinese National Medical Products Administration and certified under the EU Medical Device Regulation; it combines a micromechanical air pump, the TruBP pressure module, and temperature, photoplethysmography (PPG), and electrocardiography sensors to deliver continuous perioperative monitoring of body temperature, HR, oxygen saturation, and blood pressure. To limit motion- and wear-related artifacts, the watch uses an upgraded TruSeen 5.0+ optical system with 8 circumferential photodiodes and dual LEDs, a Fresnel film, and a curved 2.5-D sapphire back with physical vapor deposition electrodes that improve skin contact; abnormal strap loosening or compression triggers an automatic hardware repeat-measurement and refusal protocol. Raw signals were processed in real time by a HiFi-Encoder deep learning pipeline that performs multichannel enhancement and concurrent time and frequency noise suppression, discarding any frame whose signal-quality index falls below 0.8; under vigorous movement, this yields 98% accuracy for HR deviations ≤10 beats per minute (bpm) and 95% for deviations ≤5 bpm, and the same signal-quality index filter is available to downstream analyses via Huawei Health Kit. Device accuracy had been confirmed against ANSI/AAMI/ISO 81060-2:2018 criteria, while the “Huawei Heart Study” (>3.2 million participants) demonstrated a 92.8% positive predictive value for atrial fibrillation (AF) detection, a result incorporated into the 2020 European Society of Cardiology AF guidelines, collectively supporting the WATCH D’s capability to identify and suppress artifacts and to provide clinically reliable physiological data.

### Collection of Wearable Data

The data recorded by the wearable device included body temperature, skin temperature, HR, oxygen saturation, sleep quality score, shallow sleep duration, deep sleep duration, dream duration, awake duration, daytime sleep duration, total sleep duration, step volume, distance movement, and calorie consumption. For safety considerations, the blood pressure measurement function on the wearable device was disabled by technicians from the backend.

Each participant was assigned a unique application account to access HUAWEI’s developer application programming interface. Measurement data were transmitted to the application via Bluetooth, aggregated through the application programming interface backend, and deidentified before being stored on a secure network drive within the Tianhe-2 supercomputer, supported by the National Supercomputer Center in Guangzhou.

### Collection of Electronic Health Record

Patients baseline characteristics, tumor characteristics, surgical information, and perioperative characteristics were included. Baseline characteristics comprised age, sex, BMI, smoking status, and consumption history. Tumor characteristics included pathological type, tumor location, tumor size, and tumor-node-metastasis stage. Surgical information encompassed American Society of Anesthesiologists grade, operation time, surgical procedures, anesthesia method, lymph node dissection, and intraoperative bleeding.

Postoperative characteristics included length of hospital stay, duration of chest tube placement, pleural drainage volume, body temperature, oxygen saturation, HR, amount of postoperative pain medication, frequency of pain medication, administration via pain medication, administration method, and visual analog score for pain and postoperative complications.

### Collection of ePROs Data

ePROs included 9 symptoms, which is described in a former study [12], related on a scale from 0 (no symptom) to 10 (the most severe symptom). These symptoms were pain, cough, shortness of breath, restless sleep, fatigue, drowsiness, walking difficulties, and limitation of activities and distressed.

A link to the survey was sent each night at 7 PM during hospitalization. Automatic reminders were sent every 30 minutes thereafter if the questionnaires were not completed. If the survey was not completed by 10 PM, the investigator would remind the patients to complete it. All collected data were aggregated and stored using the same strategy as described earlier.

### Time-Series Data Processing

Given the considerable variations in the duration of wearing the wearable device during the perioperative period among different patients, we normalized the time scale. Specifically, we set the start time of surgery as the origin time, with the preoperative period as negative time and the postoperative period as positive time.

A large amount of time-series data could be obtained using wearable devices, including body temperature, skin temperature, HR, and oxygen saturation. Multiple measurements could be recorded within a single minute to enhance the analyzability of the time-series data, and to further reduce the likelihood of potential false positives and false negatives arising from spurious signals, we averaged all measurements obtained within each minute and used these aggregated values in downstream analyses.

### Statistical Analysis

Mean and SD or median and range were used to describe continuous data, while categorical data were presented as numbers and percentages. The agreement between the reference method and the wearable device was assessed using the Bland-Altman analysis [13]. To evaluate the feasibility and reliability between the wearable device and the reference method, Bland-Altman analysis was conducted, which is widely regarded as the gold standard for consistency assessment. This method quantifies the systematic bias and limits of agreement between 2 quantitative measurement methods, thereby providing statistical support for evaluating the reliability of the wearable device system. Subgroup analyses evaluating the reliability and feasibility of the digital device were conducted among patients with and without significant postoperative complications. Threshold analysis based on the ePROs score was used to evaluate relationships between different modules data.

Wearable devices can bridge gaps in patient monitoring within the traditional episodic care model. We analyzed the occurrences of outlier detections during periods lacking nursing supervision and visualized these occurrences as line graphs. After aggregating the raw high-frequency measurements into 1-minute averages, we defined outliers on the basis of these minute-averaged values: a minute-averaged body temperature >37 °C or <36 °C; a minute-averaged HR >90 bpm or<60 bpm; and a minute-averaged oxygen saturation <95%. Differences between groups were compared using Student *t* test (2-tailed), chi-squared test, or Fisher exact test.

All data preparation, analyses, and visualization were performed using R (version 4.0.5; The R Core Team, R Foundation for Statistical Computing). We deemed *P* values <.05 were considered statistically significant.

## Results

### Participant Demographics and Baseline Data

**Figure 1:**
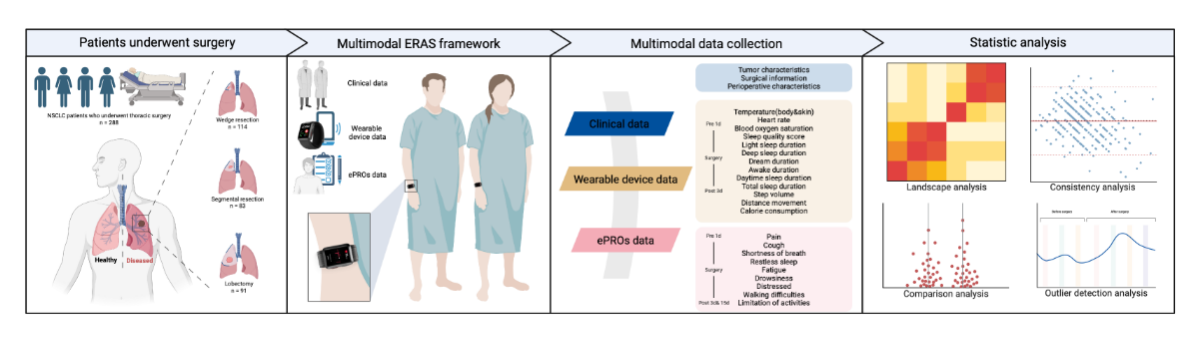
Schema of overall study design. A total of 288 patients were prospectively involved in this study. Multimodal data, including electronic health records (EHRs), wearable devices, and electronic patient-reported outcomes (ePROs), were collected, and multimethod statistical analysis was applied. ERAS: enhanced recovery after surgery. See Multimedia Appendix 1 for a high resolution version of this figure.

**Figure 2:**
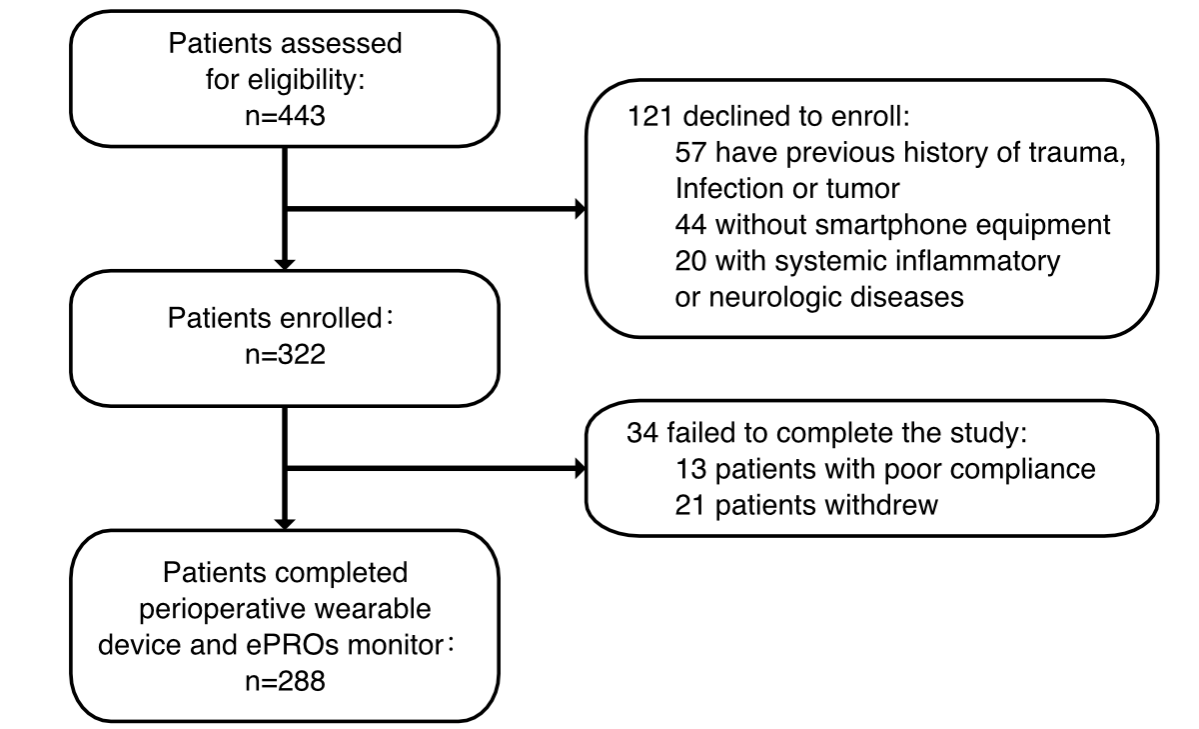
Patient recruitment. The flowchart illustrates the recruitment process and inclusion and exclusion criteria, and included participants underwent multimodal digital device monitoring. ePRO: electronic patient-reported outcome. See Multimedia Appendix 1 for a high resolution version of this figure.

A schematic overview of the study is presented in Figure 1. As presented in the flowchart diagram in Figure 2, 288 patients consented to participate in the study, including 174 female patients and 114 male patients with a median age of 56 (range 24-82) years. Baseline characteristics of the participants were presented in Table 1. At baseline, most patients underwent segmentectomy (n=203, 70.5%), while 85 (29.5%) underwent lobectomy. In total, 37 (12.8%) patients were former smokers, and 27 (9.4%) patients had a history of alcohol consumption. A total of 83 (28.8%) patients reported preoperative comorbidities, including hypertension, diabetes mellitus, chronic obstructive pulmonary disease, arrhythmia, coronary artery disease, and valvular heart disease. No intraoperative complications or surgical conversions were reported.

**Table 1:** Baseline characteristics of enrolled participants (N=288).

Characteristic			Value
**Sex, n (%)**			
	Female	174 (60.4)	
	Male	114 (39.6)	
Age (y), median (range)			56 (24-82)
**BMI (kg/m^2^), n (%)**			
	<18.5	20 (6.9)	
	18.5-24.9	201 (69.8)	
	≥25	67 (23.3)	
**Smoking history, n (%)**			
	Former smoker	37 (12.8)	
	Never smoker	251 (87.2)	
**History of alcohol use, n (%)**			
	Current alcohol use	27 (9.4)	
	Never drank alcohol	261 (90.6)	
**Preoperative comorbidities, n (%)**			
	Hypertension	29 (10.1)	
	Diabetes mellitus	23 (8)	
	COPD^a^	17 (5.9)	
	Arrhythmia	7 (2.4)	
	CAD^b^	4 (1.4)	
	Valvular heart disease	3 (1)	
**Family history of cancer, n (%)**			
	Yes	46 (16)	
	No	242 (84)	
**Surgical position, n (%)**			
	LUL^c^	70 (24.3)	
	LLL^d^	33 (11.5)	
	RUL^e^	73 (25.3)	
	RML^f^	19 (6.6)	
	RLL^g^	49 (17)	
	LUL+LLL	17 (5.9)	
	RUL+RML	6 (2.1)	
	RUL+RLL	16 (5.6)	
	RML+RLL	3 (1)	
	RUL+RML+RLL	2 (0.7)	
**Surgical method, n (%)**			
	Lobectomy	85 (29.5)	
	Segmentectomy	203 (70.5)	
**Anesthesia method, n (%)**			
	Spontaneous ventilation	116 (40.3)	
	Mechanical ventilation	172 (59.7)	
**Tumor stage, n (%)**			
	Tis	50 (17.4)	
	1	198 (68.8)	
	2	32 (11.1)	
	3	7 (2.4)	
	4	1 (0.3)	
**Node stage, n (%)**			
	Nx	14 (4.9)	
	0	254 (88.2)	
	1	11 (3.8)	
	2	8 (2.8)	
	3	1 (0.3)	
**Metastasis stage, n (%)**			
	0	285 (99)	
	1	3 (1)	
**ASA^h^ grade, n (%)**			
	I	5 (1.7)	
	II	241 (83.7)	
	III	42 (14.6)	
Surgical time (h), median (range)			1.50 (0.40-7.05)
Anesthesia time (h), median (range)			3.08 (1.35-8.15)
Intraoperative blood loss (mL), median (range)			10.00 (0-1300.00)

^a^COPD: chronic obstructive pulmonary disease.

^b^CAD: coronary artery disease.

^c^LUL: left upper lobe.

^d^LLL: left lower lobe.

^e^RUL: right upper lobe.

^f^RML: right middle lobe.

^g^RLL: right lower lobe.

^h^ASA: American Society of Anesthesiologists.

### Mapping Patient Vital Status and Recovery During Perioperative Vital Signs and Recovery

**Figure 3:**
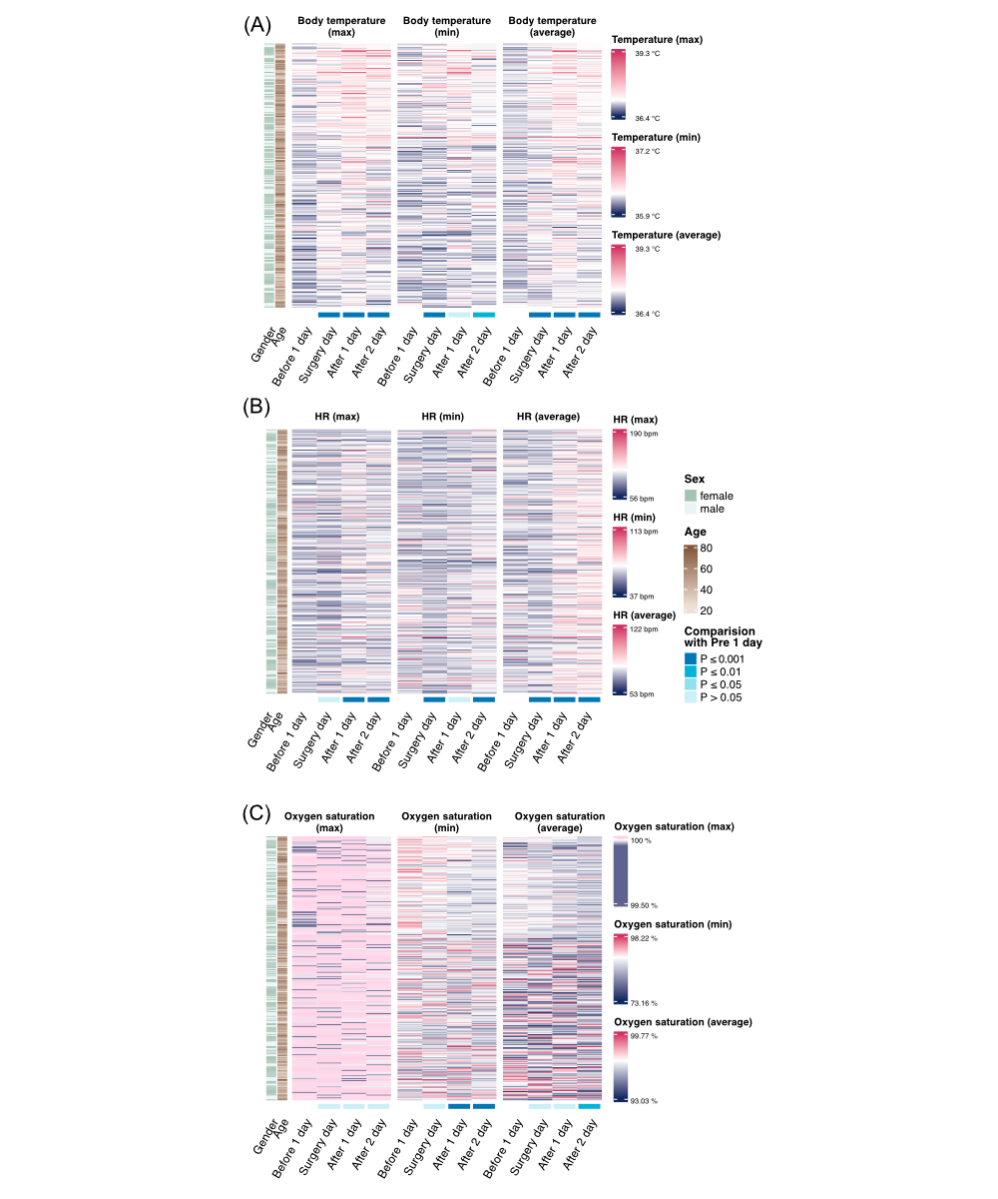
Heatmap illustrates the patients’ perioperative body temperature, heart rate (HR), and oxygen saturation using wearable device data. (A) body temperature, (B) HR, and (C) oxygen saturation. Each parameter is displayed with its maximum, minimum, and average values. The sex and age of each patient are annotated and displayed alongside the heatmap. NS: not significant. See Multimedia Appendix 1 for a high resolution version of this figure.

**Figure 4:**
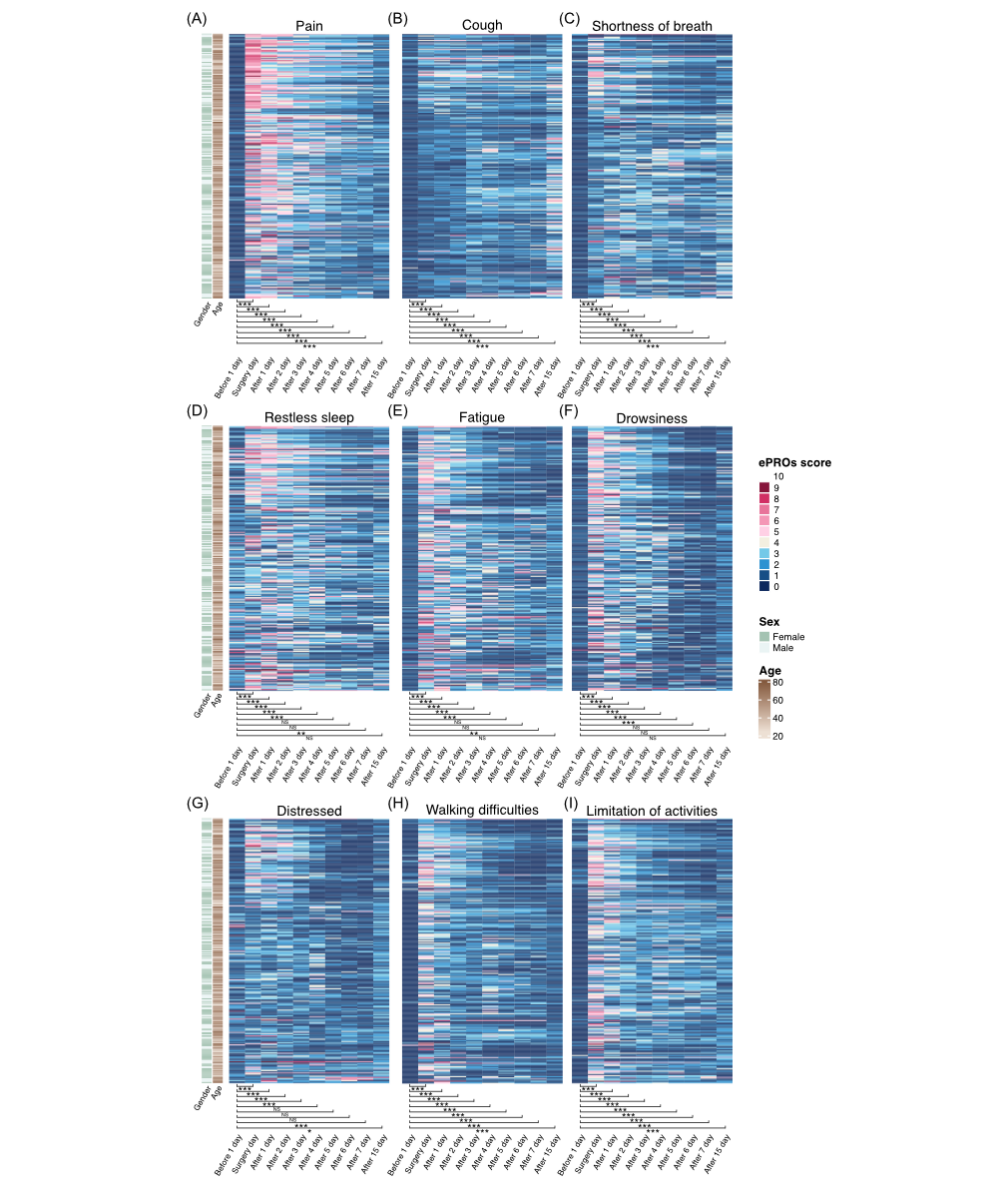
The heat map illustrates the patients’ perioperative recovery status using electronic patient-reported outcomes (eRPOs) data. (A) Pain, (B) cough, (C) shortness of breath, (D) restless sleep, (E) fatigue, (F) drowsiness, (G) distressed, (H) walking difficulties, and (I) limitation of activities. NS: not significant. See Multimedia Appendix 1 for a high resolution version of this figure.

Figures 3 and 4 demonstrate the utility of integrating wearable device data and ePROs to provide a comprehensive, real-time assessment of perioperative recovery. This analysis highlights the advantages of continuous monitoring in detecting critical physiological changes and tracking patient-reported symptoms during thoracic surgery recovery.

In the analysis of wearable device data, both body and skin temperatures showed consistent increases after surgery, indicating inflammatory or healing responses (Figure 3A and Multimedia Appendix 2). HR demonstrated a clear upward trend, with statistically significant increases in maximum and average HR on the first and second postoperative days, highlighting the physical stress of surgery (Figure 3B). Oxygen saturation levels displayed a significant overall decline, especially in minimum and average values during the perioperative period, suggesting potential postoperative respiratory compromise that warrants close monitoring (Figure 3C). Early improvements in physical mobility, as evidenced by significant increases in step count, exercise distance, and calorie expenditure on the first postoperative day (Multimedia Appendix 2), underscore the rapid physical recovery promoted by ERAS protocols. Importantly, sleep quality significantly deteriorated on the day of surgery and the first postoperative day, reflecting the immediate postoperative impact on psychological recovery (Multimedia Appendix 2).

A total of 288 ePROs questionnaires were collected and analyzed. Figures 4A-4I showed that symptoms such as pain, cough, shortness of breath, and activity limitations peaked on the day of surgery and significantly improved within the first week and by day 15. This gradual symptom alleviation aligns with the expected recovery timeline, but the persistent challenges with sleep and fatigue underscore the need for tailored recovery interventions. The combination of wearable devices and ePROs provides a robust framework for perioperative monitoring, offering both objective physiological data and subjective patient feedback.

We also calculated the median and IQRs of ePRO scores during the perioperative period. The results suggested a pattern consistent with the objective trajectory of postoperative recovery. Taking pain as an example, the median pain score and IQR were 0 (IQR 0-0) on the day before surgery. The score peaked on the day of surgery, with a median of 5 (IQR 3-7), and gradually decreased starting from the second postoperative day, remaining at a low level. By the seventh postoperative day, the median pain score was 1 (IQR 1-2), and it further decreased by the 15th postoperative day, with a median of 1 (IQR 0-2; Multimedia Appendix 3).

### Evaluation of Agreement Between Wearable Devices and Electronic Health Record

**Figure 5:**
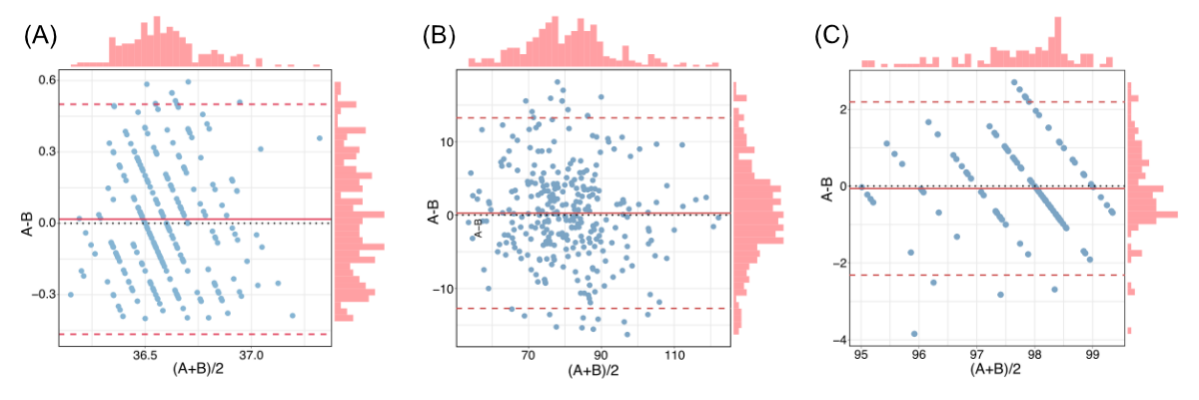
Bland-Altman plot shows the agreement between digital devices and electronic health records (EHRs) in all participants. (A) Body temperature, (B) heart rate (HR), and (C) oxygen saturation. See Multimedia Appendix 1 for a high resolution version of this figure.

**Figure 6:**
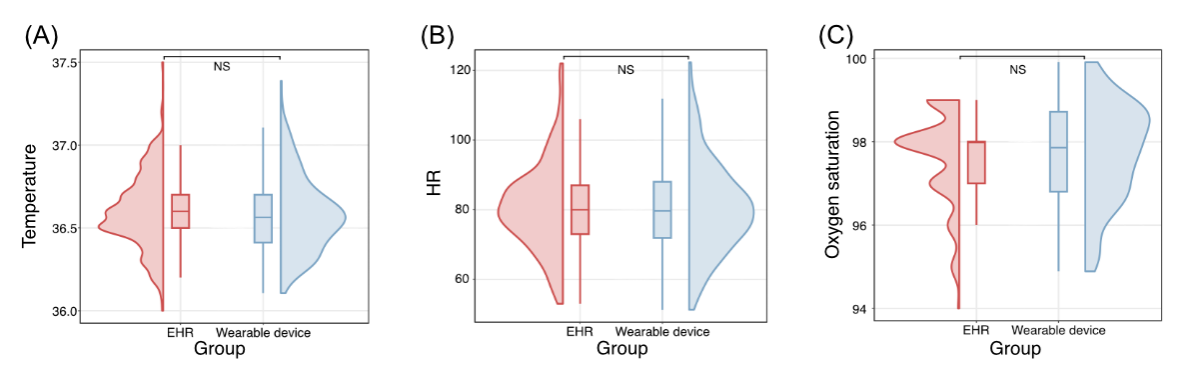
Raincloud plot shows the comparison between digital devices and electronic health records (EHRs) in all participants. (A) Body temperature, (B) heart rate (HR), and (C) oxygen saturation. See Multimedia Appendix 1 for a high resolution version of this figure.

A Bland-Altman analysis was conducted to assess the agreement between wearable device data and electronic health record (EHR; episodic care model) for body temperature, HR, and oxygen saturation. For body temperature, the analysis revealed a minimal bias of 0.02 °C (95% CI –0.01 °C to 0.05 °C), with limits of agreement ranging from –0.47 °C (95% CI –0.52 °C to –0.41 °C) to 0.50 °C (95% CI 0.45 °C to 0.55 °C), indicating strong consistency between the 2 methods (Figure 5A). The agreement for HR showed a bias of 0.26 bpm (95% CI –0.49 bpm to 1.01 bpm), with limits of agreement between –12.71 bpm (95% CI –14.00 bpm to –11.43 bpm) and 13.24 bpm (95% CI 11.95 bpm to 14.53 bpm), reflecting slightly broader variability but overall reasonable alignment between the wearable device and EHR (Figure 5B). For oxygen saturation, the bias was –0.06% (95% CI –0.27% to 0.15%), with limits of agreement from –2.31% (95% CI –2.68% to –1.95%) to 2.19% (95% CI 1.83% to 2.56%), indicating close alignment between the 2 measurement methods (Figure 5C). Paired sample *t* tests confirmed a high level of agreement in the mean values for body temperature (Figure 6A), HR (Figure 6B), and oxygen saturation (Figure 6C), further supporting the reliability of wearable devices in perioperative monitoring. This analysis highlights that wearable devices provide accurate and clinically relevant measurements.

### Continuous Vital Signs Monitoring: Bridging Gaps in Perioperative EHR Systems

Perioperative records in EHR systems often suffer from discontinuities, with significant temporal gaps in vital sign data. To address this issue, we conducted a systematic review to evaluate the benefits of wearable devices for continuous, 24-hour monitoring of vital signs. Table 2 summarizes the comparative performance of wearable devices in this context.

**Table 2:** Comparative performance and outliers between electronic health records (EHRs) and wearable devices.

		EHR	Wearable device	*P* value
**Body temperature (times)**				
	Total number per patient	10.00	14,219.04	<.001
	Total number per patient per day	3.08	4440.69	<.001
	Outliers number per patient	0.23	2707.47	<.001
	Outliers number per patient per day	0.07	845.56	<.001
**He** **art rate** **(times)**				
	Total number per patient	10.19	4293.75	<.001
	Total number per patient per day	2.65	1339.16	<.001
	Outliers number per patient	2.08	964.96	<.001
	Outliers number per patient per day	0.54	300.96	<.001
**Oxygen saturation (times)**				
	Total number per patient	6.83	6924.67	<.001
	Total number per patient per day	1.77	2057.24	<.001
	Outliers number per patient	0.17	2644.67	<.001
	Outliers number per patient per day	0.04	785.70	<.001

During hospitalization, body temperature, HR, and oxygen levels were measured an average of 3.08, 2.65, and 1.77 times per patient per day, respectively, during ward rounds, leading to 10.00, 10.19, and 6.83 measurements per patient over the course of their stay. In contrast, wearable devices recorded these parameters continuously, providing an average of 4440.69, 1339.16, and 2057.24 measurements per patient per day, with total readings reaching 14,219.04, 4293.75, and 6924.67 times.

The difference in detection sensitivity was particularly notable when comparing outlier detection. For body temperature, the ward rounds identified 0.07 times outliers per patient, per day, whereas the wearable devices detected significantly more outliers, averaging 845.56 times per patient per day. Similarly, HR outliers were recorded at a rate of 0.54 times per patient per day through ward rounds, compared to 300.96 times detected by the wearable devices. For oxygen saturation levels, ward rounds captured 0.04 times outliers per patient per day, while wearable devices identified 785.70 times outliers.

This comparison underscores the advantage of wearable devices in providing continuous, real-time vital sign monitoring, filling the gaps left by intermittent EHR-based records, and enabling earlier detection of critical physiological changes.

### Typical Case Presentation and Comparison

To provide a clearer and more intuitive comparison between episodic care methods and digital devices, we sampled 2 typical cases and visualized their perioperative body temperature, HR, and oxygen saturation levels.

**Figure 7:**
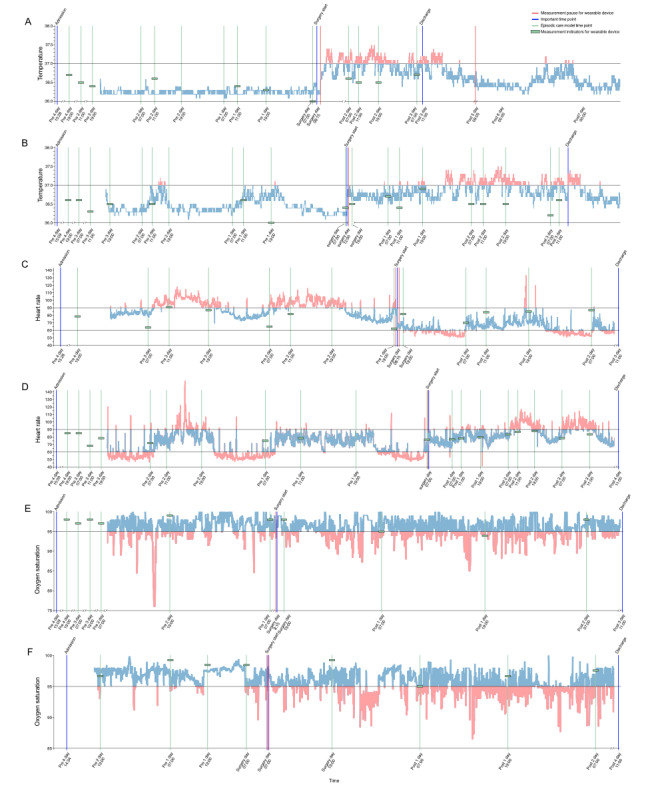
Typical case presentation of perioperative continuous monitoring and comparison between episodic care method and digital device. (A) Perioperative body temperature trends of case 1, (B) perioperative body temperature trends of case 2, (C) perioperative heart rate (HR) trend of case 1, (D) perioperative HR trend of case 2, (E) perioperative oxygen saturation trend of case 1, and (F) perioperative oxygen saturation trend of case 2. See Multimedia Appendix 1 for a high resolution version of this figure.

Figures 7A and 7B illustrated the perioperative body temperature trends for both patients. Preoperatively, the medical team assessed body temperature 11 and 17 times, respectively, for each patient, with all readings within normal ranges. Wearable device data closely mirrored these values, with most preoperative measurements also falling within normal limits. Notably, patient #1 maintained a more stable body temperature compared to patient #2, who experienced 2 transient spikes. However, the wearable device detected additional outliers that were missed by conventional monitoring, particularly in the intervals between routine checks.

Figures 7C and 7D depict the perioperative HR monitoring. The medical team recorded HR 12 and 16 times for each patient, respectively, and noted one outlier in the male patient. In contrast, the wearable device revealed time-specific fluctuations in HR that were not captured by conventional monitoring. Activities such as departmental visits occasionally led to missed measurements, an issue addressed by the continuous monitoring of the wearable device. For instance, in the selected female patient, measurements were missed at 11 AM and 7 PM on April 12, 2023, during which significant HR fluctuations occurred, as revealed by the wearable device.

Figures 7E and 7F show paired perioperative oxygen saturation monitoring. The medical team measured oxygen saturation levels 10 and 8 times for each patient, again noting an outlier in the male patient. The wearable device data showed high consistency with these traditional measurements while also providing continuous, uninterrupted data.

### Assessing the Correlation Between Wearable Metrics and ePROs Scores

To explore the potential association between data from wearable devices and ePROs, we conducted a threshold analysis comparing wearable parameters between 2 subgroups, categorized based on the overall ePROs score and 9 subindexes.

Multimedia Appendix 4 summarizes the results of the threshold analysis based on the overall ePROs score. Notably, patients in the high-score group exhibited significantly higher extreme and mean values of HR, as well as lower minimum and mean oxygen saturation levels, compared to those in the low-score group. In addition, the high-score group showed elevated maximum body temperature and poorer sleep quality during the perioperative period. These findings suggest a strong association between elevated physiological stress, as detected by the wearable device, and higher self-reported symptom burden from ePROs.

Further analysis based on the 9 subindexes, detailed in Multimedia Appendix 5, revealed significant differences in extreme and mean values of body temperature, HR, and oxygen saturation levels between subgroups. These results underscore the association of wearable metrics in reflecting the severity of ePROs, providing valuable insights for more personalized perioperative management.

### Reported Feasibility and Acceptance of Digital Device

Upon completing the study, all patients were interviewed to assess their experience with the ePROs and wearable devices. Table 3 presents the summarizations of their feedback. Overall, patients reported a positive experience, stating that both the wearable device and the ePRO questionnaires were easy to use, and the device was comfortable to wear and simple to maintain. The primary feedback centered on enhancing the functionality of the wearable device. Patients suggested improvements to the software interface for better usability and recommended adding a blood pressure monitoring feature to further expand the device’s capabilities.

**Table 3:** Feedback regarding the digital device from patient and medical team interviews.

Themes	Interview response
Overall experience	“It has a positive effect on my preoperative status assessment and postoperative recovery.” “It is non-invasive, I just need to wear the watch as usual and complete the electronic questionnaire.” “It is waterproof, I can even keep wearing it while taking a shower.” “I don’t need to charge it frequently, once or twice a week is sufficient, which is very convenient.” “I’m satisfied, medical team can better understand my condition through the wearable device and ePROs, which makes me feel valued.”
Device comfort	“It looks like a watch for daily wear.” “There are different types of watchbands to choose, offering a comfortable, barely noticeable wear.” “It is very lightweight, and I wear it while sleeping without any impacts.” “I can easily fill the questionnaires on my phone, simply selecting options based on my condition. Only takes a few minutes to complete.” “The questionnaire comes with reminder notifications on my phone, ensuring that I don’t forget to complete it.”
Problems and improvements to the device	“Plastic watchbands tend to cause swearing when worn outdoors in the summer.” “Is it possible to add a blood pressure monitoring feature?” “Is it possible to use an infinitely adjustable watchband?” “Is it possible to combine the wearable device’s software with the questionnaire app into one? Download multiple apps can be quite frustrating.”
Useful for the medical team?	“Yes, it would greatly assist the medical team in supplementing critical data amidst their busy work schedules.” “Yes, it can be more objectively reflect the patient’s perioperative vital status.” “Yes, it also serves as a motivation for patients. When patients wear the device and complete the ePROs questionnaires, it encourages them to be more proactive in order to achieve feedback and outcomes.”

### Evaluation of the Digital Device in Patients With and Without Postoperative Complications

Postoperative complications were recorded in 25 (8.7%) out of 288 patients. Of these 25 patients, 13 (52%) experienced air leaks, 6 (24%) developed sustained high fever, 5 (20%) had persistent pleural drainage, and 1 (4%) exhibited poor lung re-expansion. The remaining 263 (31.3%) patients did not report any recordable postoperative complications. The baseline characteristics of patients with postoperative complications were summarized in Multimedia Appendix 6.

Therefore, we performed the subgroup analyses based on patients with postoperative complications. The results of the Bland-Altman analysis demonstrated consistency with the overall findings. The body temperature revealed a minimal bias of –0.02 °C (95% CI –0.33 °C to 0.29 °C), with limits of agreement ranging from –0.33 °C (95% CI –0.44 °C to –0.22 °C) to 0.30 °C (95% CI 0.18 °C to 0.40 °C; Multimedia Appendix 7). The agreements for HR showed a bias of 1.65 bpm (95% CI –1.21 bpm to 4.51 bpm), with limits of agreement between –11.95 bpm (95% CI –16.66 bpm to –7.24 bpm) and 15.24 bpm (95% CI 10.53 bpm to 19.95 bpm; Multimedia Appendix 6). For oxygen saturation, the bias was 0.27% (–0.24% to 0.78%), with limits of agreement from –2.14% (95% CI –2.98% to –1.31%) to 2.68% (95% CI 1.85% to 3.52%; Multimedia Appendix 8). The results of the paired sample *t* test demonstrated a high level of consistency between digital device and EHR measurements in patients with postoperative complications (Multimedia Appendix 7).

We also conducted a subgroup analysis of patients without postoperative complications; their baseline characteristics were summarized in Multimedia Appendix 9. The results demonstrated a high degree of agreement between digital devices and EHR measurements (Multimedia Appendices 10 and 11).

In summary, the results of the subgroup analysis demonstrated a high level of consistency with the overall findings, thereby confirming the robustness of the study’s main results. Furthermore, these findings reinforced that the digital device was capable of effectively capturing clinically relevant vital information even in complex clinical settings, supporting its broad feasibility and reliability for clinical application.

## Discussion

### Principal Findings

This is the first study to investigate the feasibility and reliability of using digital devices, including wearable devices and ePRO questionnaire, in the perioperative management of patients with lung cancer who underwent thoracic surgery. In this study, perioperative recovery was monitored through digital devices. Our results indicated that multimodal data could reflect patients’ perioperative status objectively and provide rapid, sensitive outlier detection. This innovation can assist the medical team in optimizing recovery trajectories and achieving the goals of ERAS in the context of the MITS era.

Using prospectively collected data, we systematically mapped the perioperative recovery trajectory of patients with lung cancer who underwent thoracic surgery. Comparison across days revealed significant improvements in the vast majority of parameters, underscoring their critical role in recovery. Notably, the duration of shallow sleep, deep sleep, dreaming, total sleep, and day sleep, as recorded by the wearable device, showed no significant differences across days. However, the sleep quality score significantly improved during hospitalization. This discrepancy aligns with the restless sleep and drowsiness reported via ePROs, which reflect patients’ subjective experiences, suggesting a higher level of accuracy [14,15]. Possible explanations include improved sleep structure, fewer sleep-cycle disruptions, enhanced subjective wakefulness and sleep experience, and gradual environmental adaptation. In addition, psychological changes after surgery, such as anxiety and distress, also play a role, as reflected in other ePROs-derived measures.

Although this is a noninterventional study, its potential impact on nursing care and therapeutic procedures holds significant clinical importance. Through the recovery trajectories monitored by wearable devices and ePROs, we were able to capture real-time vital signs and subjective experiences of patients, thereby providing more comprehensive information for clinical decision-making. As a self-reported tool, ePROs effectively reflect patients’ subjective experiences, such as anxiety, pain, and activity limitations, and changes in their scores can serve as triggers for interventions. Specifically, when a score in a particular ePROs measure exceeds a set threshold, the nursing team can immediately implement corresponding interventions based on the patient’s reported concerns. For example, if a patient reports high pain levels, the nursing team can provide a pain management plan according to the assessment results; if a patient’s anxiety score exceeds the threshold, a psychological evaluation and consultation can be arranged. This precision-based intervention model using ePROs enables more individualized care, preventing both overintervention and undercare, thereby more effectively supporting the patient’s recovery process. In addition, digital devices can promptly alert nursing staff when vital signs deviate from normal ranges, enabling timely interventions. The integration of ePROs and digital device monitoring data significantly enhances nursing efficiency, optimizes resource allocation, and reduces unnecessary interventions. Future interventional studies will further explore how these monitoring data can be integrated into routine clinical workflows to improve perioperative recovery outcomes and enhance nursing care quality.

Wearable devices are gaining attention in monitoring patients, and the health population has garnered significant attention. These devices are equipped with various sensors and chips designed to track a wide range of data, including HR sensors, accelerometers, gyroscopes, temperature sensors, oxygen saturation sensors, electrode sensors, and ambient light sensors. While their convenience is widely recognized, concerns regarding reliability and data security remain [16,17]. In this study, we conducted pairwise comparisons of body temperature, HR, and oxygen saturation obtained from EHR and wearable devices, respectively. Bland-Altman agreement analysis and paired sample *t* test analysis demonstrated a high degree of consistency between the 2 modules' data, supporting the reliability, validity, and clinical utility of wearable devices. Subgroup analysis in patients with and without postoperative complications also showed consistency between overall results, further confirming the feasibility and reliability of the proposed digital device approach in this study. This is consistent with previous studies [18-21], including a systematic review by Germini et al [22], which confirmed the accuracy and acceptability of wrist-wearable activity trackers. This review included 65 studies, assessing 14 accuracy-related outcomes. Step count, HR, and energy expenditure were the most frequently evaluated. Fitbit Charge and Fitbit Charge HR (Fitbit Inc) showed the highest accuracy for step counts, while the Apple Watch (Apple Inc) performed better for HR. Acceptability, measured by data availability and wearing time, revealed that Fitbit Charge HR, Fitbit Flex 2, and Garmin Vivofit (Garmin Ltd) had the highest data availability, whereas GENEActiv (Activinsights Ltd) and FuelBank (Nike Inc) had the longest wearing time. Although previous studies, as mentioned earlier, have extensively demonstrated the accuracy and reliability of wearable devices, we acknowledge that in this study, we accepted the credibility of data collection provided by Huawei Technologies and did not conduct further independent validation of these data. Nevertheless, it is important to emphasize that quality assurances provided by manufacturers are not fully equivalent to rigorous independent data validation conducted in academic research. Therefore, the interpretation of our results should consider this specific limitation.

In this study, the wearable device estimated core body temperature by measuring peripheral skin temperature at the wrist. During the development phase, the research team established a mapping model by simultaneously collecting data on both core and skin temperatures. This model was further optimized using physiological and environmental parameters such as HR and ambient temperature. After the stability of the model was validated, it was integrated into the device’s algorithm, enabling accurate real-time estimation of core body temperature based on skin temperature readings. In addition, the temperature sensor underwent calibration and performance testing under various environmental conditions to ensure measurement accuracy.

Continuous monitoring offers distinct advantages in perioperative management. In contrast to traditional EHRs, which capture vital signs at discrete time points, wearable devices provide real-time, continuous monitoring and reduce the likelihood of missing critical clinical changes. A key advancement in this area is the development of PPG technology, which has made HR monitoring a reliable and established feature of wearable devices [23,24]. A recent review in the *New England Journal of Medicine* summarized and provided an outlook on wearable digital health technologies for monitoring cardiovascular conditions. The gradual integration of wearable devices into cardiovascular care can support continuous monitoring of patients’ vital signs and other physiological parameters, enabling rapid remote monitoring and management. This approach aims to facilitate early detection of disease deterioration, improve perioperative management, and reduce readmission rates [25]. Guo et al [26] evaluate the effectiveness of PPG technology integrated into wearable devices for detecting AF in a large population-based cohort across China. This study included 187,912 participants who monitored their pulse rhythm using a HUAWEI smartwatch for at least 14 days. Results showed that 0.23% (424/187,912) of the participants received a “suspected AF” notification, with 87% of cases confirmed as AF through clinical evaluation, yielding a positive predictive value of 91.6%. The study concluded that continuous home monitoring with PPG-based wearable devices is a feasible and effective method for early AF detection, enabling timely interventions to reduce AF-related complications. On the basis of these findings, wearable digital health technologies have been recommended for cardiovascular monitoring in the 2020 European Society of Cardiology guidelines [27].

In the perioperative management within the surgical community, wearable devices have shown promise across various surgical disciplines. Beqari et al [28] conducted a pilot study with 56 patients who had undergone cardiothoracic surgery to evaluate the effectiveness of the “NightSigal” algorithm. Patients were asked to wear a Fitbit watch for at least 1 week preoperatively and up to 90 days postoperatively to collect biometric data. The algorithm detected 17 out of 21 postoperative events, with a median detection time of 2 days before symptom onset. Sensitivity and specificity were 81% and 75%, respectively. Ghomrawi et al [29] assessed the use of Fitbit to monitor the postoperative complications in patients who have undergone pediatric appendectomy. The combined predictive ability for complex appendicitis was an area under the curve of 0.80, compared to 0.70 for simple appendicitis. Kane et al [30] examined activity monitors for predicting 30-day readmission by tracking activity levels before and after surgery. Readmitted patients showed lower return to baseline activity compared to nonreadmitted patients. After adjusting for cofounders, multivariable analysis demonstrated that returning to baseline activity was associated with a reduced risk of 30-day readmission. The model had a sensitivity of 75% and a specificity of 74%.

Despite the advances seen in other surgical fields, the utility of wearable devices in patients with lung cancer undergoing thoracic surgery remains underexplored. Our findings revealed that outliers in vital signs, such as fluctuations in body temperature, HR, oxygen saturation levels, and other key metrics, occurred between the time points of EHR monitoring. Implementing robust continuous monitoring and alarm mechanism could enable the medical team to better track patients’ baseline vital signs, thereby facilitating ERAS protocols.

Effective outlier detection in this study relied on a 2-tier strategy that integrated device-embedded safeguards with protocol-level quality control. First, at the hardware–algorithm tier, the wrist-worn HUAWEI WATCH D combined (1) an upgraded TruSeen 5.0+ optical module—8 circumferential photodiodes, dual LEDs, a Fresnel film, and a curved 2.5-D sapphire or physical vapor deposition back—plus the TruBP pressure system [31], and (2) a HiFi-Encoder deep learning pipeline that discards frames whose signal-quality index is <0.8; together these measures cut motion-related signal loss to one-quarter of that in the previous generation and yielded HR accuracies of 98% (≤10 bpm) and 95% (≤5 bpm) during vigorous movement. A dual-mode PPG-electrocardiography architecture driven by a high-performance chip was trained on large baseline datasets collected in rest, exercise, commuting, sleep, heat, and underwater conditions; it has already demonstrated a 92.8% positive predictive value for AF detection in >3.2 million participants and is cited in the 2020 European Society of Cardiology AF guidelines [27], while independent testing confirmed full compliance with ANSI/AAMI/ISO 81060-2:2018 [32]. The device is further supported by class II approval from China’s National Medical Products Administration and European Union Medical Device Regulation certification. Second, at the study-design tier, intensive patient training and ward-level adherence checks led to exclusion of 13 poorly compliant participants; temperature, HR, and oxygen-saturation readings were cross-validated against EHR values to confirm accuracy; and continuous data were aggregated into 1-minute maxima, minima, and means, with outliers defined as minute-averaged temperature >37°C or <36°C, HR >90 or <60 bpm, or oxygen saturation <95%. This minute-level preprocessing attenuated sporadic spikes and focused analyses on sustained, clinically meaningful deviations. Collectively, the 6 elements—noise-reduction hardware, real-time AI filtering, extensive algorithm training, external validation and regulation, adherence management, and time-series preprocessing—provided a robust framework for distinguishing true physiological outliers from artifacts, thereby reinforcing the reliability and clinical applicability of our perioperative monitoring results.

The benefits of integrating EHRs with wearable devices are gradually becoming more apparent. Building on this foundation, we further incorporated ePROs to develop a multimodule perioperative monitoring network. Previous studies have demonstrated that patient reported outcomes (PROs) can effectively capture various aspects of a patient’s perioperative status, such as sleep, fatigue, and drowsiness [33]. Dai et al [8] conducted a multicenter, randomized controlled trial demonstrating the feasibility and effectiveness of PROs-based symptom management in patients who underwent lung cancer surgery. In the early recovery phase, PROs-based groups experienced significantly fewer symptom threshold events at discharge within 4 weeks after discharge, with a lower incidence of complications, highlighting their potential to reduce symptom burden and complication rates. Earlier this year, Dai et al [9] updated the long-term follow-up results, showing that ePROs groups had a lower symptom burden and higher physical and emotional function scores over a follow-up period of 1 to 12 months compared to usual care.

In this study, we introduced a threshold analysis to stratify patients based on the ePROs overall score and subindexes. Most vital signs and physiological parameters measured by the wearable device showed statistically significant differences between the high and low ePROs score groups, suggesting a potential relevance between various digital device modules. This highlights the advantages of using multimodal data to comprehensively assess the perioperative recovery status of patients who underwent lung cancer surgery.

Our study also highlighted the potential economic benefits of the digital device–based continuous monitoring system. By enabling early detection of complications and timely interventions, the system may shorten hospital stays and reduce associated costs. Its integration into perioperative workflows can also alleviate nursing workload and improve resource efficiency. Furthermore, the use of the HUAWEI WATCH D, a certified medical-grade wearable device covered by the National Medical Products Administration, enhances the feasibility of large-scale clinical implementation.

The acceptability, usability, and comfort of digital devices are crucial determinants of their successful clinical implementation [25]. In this study, feedback from both patients and medical staff was largely positive, with participants acknowledging the ability of digital devices to gather detailed perioperative data without disrupting daily activities or routine care. Concerns were raised regarding the stability of the devices and the potential influence of external factors, such as physical movement, on data accuracy. Addressing these challenges through future technological advancements will be essential to ensuring the reliability of wearable devices in clinical practice.

To address these limitations and further enhance the robustness of digital perioperative monitoring, our study integrated ePROs as a complementary data modality alongside wearable devices. While wearable devices continuously captured objective physiological parameters such as HR and oxygen saturation, ePROs provided direct insights into patients’ subjective symptoms and functional status, including pain, fatigue, and sleep quality. The design of combining sensor-based and self-reported data improved the overall reliability and interpretability of perioperative monitoring. Moreover, this integration supported seamless, minimally burdensome data acquisition and enabled more individualized recovery profiling. In future applications, this multimodal framework may also contribute to the development of more accurate and generalizable predictive models for surgical recovery.

There are several limitations in our study that warrant acknowledgment. First, this was a single-center, prospective, self-controlled study conducted among patients undergoing thoracic surgery, which limited the generalizability of the results to other populations. However, our findings provide a foundation for future multicenter clinical trials. Second, all patients enrolled were required to own and operate a smartphone and to have a basic understanding of the functions provided by the wearable device; while the research team made every effort to provide technical support and detailed assistance to all potentially eligible patients, we acknowledge that individuals with limited technological literacy, including some older adults, may have been excluded, which highlights the importance of future research into the usability and compliance of wearable technologies across diverse patient demographics. Third, although the 2-tier strategy of device-embedded safeguards coupled with protocol-level quality control used in this study could identify and manage most potential artifacts in the wearable-device data, we acknowledge that residual false positive and false negative readings may still have influenced the study’s results and conclusions. Fourth, although the wearable device used in this study was the first wrist-worn wearable device to receive class II medical device certification, the blood pressure measurement function required the patient to perform the measurement autonomously. This could potentially lead to frequent measurements that might affect blood circulation or result in missing or reduced data quality if measurements were not taken according to the study protocol. After careful consideration, to avoid interfering with the normal treatment process of the patients, we ultimately decided not to include blood pressure as one of the planned measurement outcomes in this study. Previous studies have demonstrated the safety and feasibility of using the HUAWEI Watch D for blood pressure measurement, which could compensate for the lack of blood pressure monitoring data in this study [21]. In the future, we will explore the clinical application of dynamic blood pressure monitoring for patients who underwent thoracic surgery using this device within a safer clinical framework. Fifth, individualized perioperative nursing interventions may have introduced outliers in the digital device–recorded data. For example, postoperative pain and the use of analgesics may have affected HR readings. Although this study used a multimodal monitoring approach to objectively reflect patient recovery from multiple perspectives and conducted a subgroup analysis to further verify the robustness of the findings, achieving real-time consistency and alignment between individualized perioperative clinical management and the parameters monitored by digital devices remains one of the key challenges for future research.

### Conclusions

Within the perioperative management of patients with lung cancer under the ERAS framework, the digital device composed of wearable devices and ePROs demonstrated feasibility and reliability in the continuous perioperative monitoring of patients who underwent lung cancer surgery. Our study offered a novel approach for multimodal, continuous perioperative monitoring, thereby laying the foundation for future research.

## Appendix

Multimedia Appendix 1 High resolution version of all figures.

Multimedia Appendix 2 Heatmap illustrated the patients’ perioperative skin temperature, activity, and sleep status using wearable device data. (A) skin temperature;(B) sleep assessment;(C) activity assessment. Each parameter was displayed with its maximum, minimum, and average values. The gender and age of each patient were annotated and displayed alongside the heatmap.

Multimedia Appendix 3 Median and Interquartile Range Distribution of electronic patients-report outcome.

Multimedia Appendix 4 Results of threshold analysis based on the ePROs overall score.

Multimedia Appendix 5 Results of threshold analysis based on the nine symptoms.

Multimedia Appendix 6 Baseline characteristic of patients with postoperative complications.

Multimedia Appendix 7 Bland-Altman plot showed the agreement between digital device and EHR in participants with postoperative complications. (A) body temperature; (B) HR; (C) oxygen saturation.

Multimedia Appendix 8 Raincloud plot showed the comparison between digital device and EHR in participants with postoperative complications. (A) body temperature; (B) HR; (C) oxygen saturation.

Multimedia Appendix 9 Baseline characteristic of patients without postoperative complications.

Multimedia Appendix 10 Bland-Altman plot showed the agreement between digital device and EHR in participants without postoperative complications. (A) body temperature; (B) HR; (C) oxygen saturation.

Multimedia Appendix 11 Raincloud plot showed the comparison between digital device and EHR in participants without postoperative complications. (A) body temperature; (B) HR; (C) oxygen saturation.

## Abbreviations

AF: atrial fibrillation
bpm: beats per minute
EHR: electronic health record
ePRO: electronic patient-reported outcome
ERAS: enhanced recovery after surgery
HR: heart rate
MITS: minimally invasive thoracic surgery
PPG: photoplethysmography
PRO: patient-reported outcome

## Data Availability

The datasets generated or analyzed during this study are not publicly available due to institutional regulations concerning patient data but are available from the corresponding author upon reasonable request.
