# Supplementary material for: Integrating a Multimodal Digital Device for Continuous Perioperative Monitoring in Patients With Lung Cancer Undergoing Thoracic Surgery: Development and Usability Study
Source: JMIR Mhealth Uhealth. 2025 Sep 16;13:e69512. doi: 10.2196/69512 (PMC12485267; doi:10.2196/69512)
Supplement: Multimedia Appendix 1 [file mhealth_v13i1e69512_app1.zip › Vector Figures Package/Figure 1.pdf]

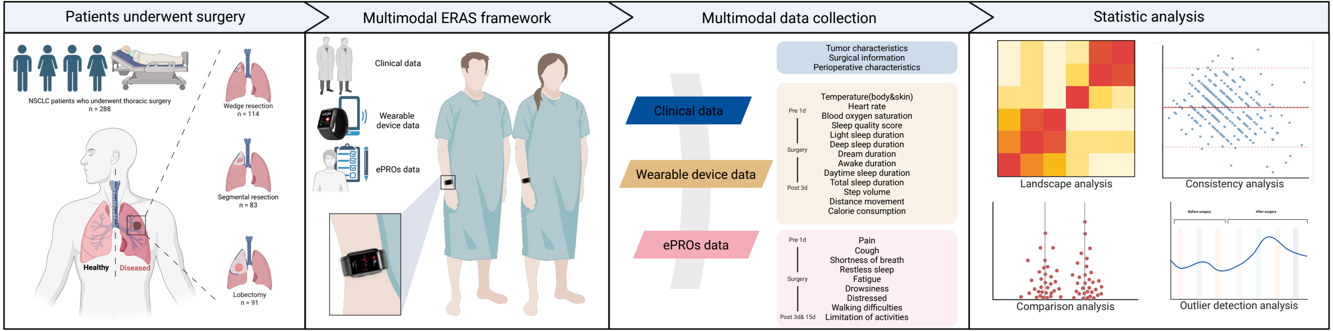

Patients underwent surgery
Multimodal ERAS framework
Multimodal data collection
Statistic analysis
NSCLC patients who underwent thoracic surgery
n = 288
Wedge resection
n = 114
Segmental resection
n = 83
Lobectomy
n = 91
Healthy
Diseased
Clinical data
Wearable device data
ePROs data
Clinical data
Wearable device data
ePROs data
Tumor characteristics
Surgical information
Perioperative characteristics
Pre 1d
Surgery
Post 3d
Temperature(body&skin)
Heart rate
Blood oxygen saturation
Sleep quality score
Light sleep duration
Deep sleep duration
Dream duration
Awake duration
Daytime sleep duration
Total sleep duration
Step volume
Distance movement
Calorie consumption
Pre 1d
Surgery
Post 3d& 15d
Pain
Cough
Shortness of breath
Restless sleep
Fatigue
Drowsiness
Distressed
Walking difficulties
Limitation of activities
Landscape analysis
Consistency analysis
Comparison analysis
Before surgery
After surgery
Outlier detection analysis
