# Supplementary figures and images for: Integrating a Multimodal Digital Device for Continuous Perioperative Monitoring in Patients With Lung Cancer Undergoing Thoracic Surgery: Development and Usability Study
Source: JMIR Mhealth Uhealth. 2025 Sep 16;13:e69512. doi: 10.2196/69512 (PMC12485267; doi:10.2196/69512)

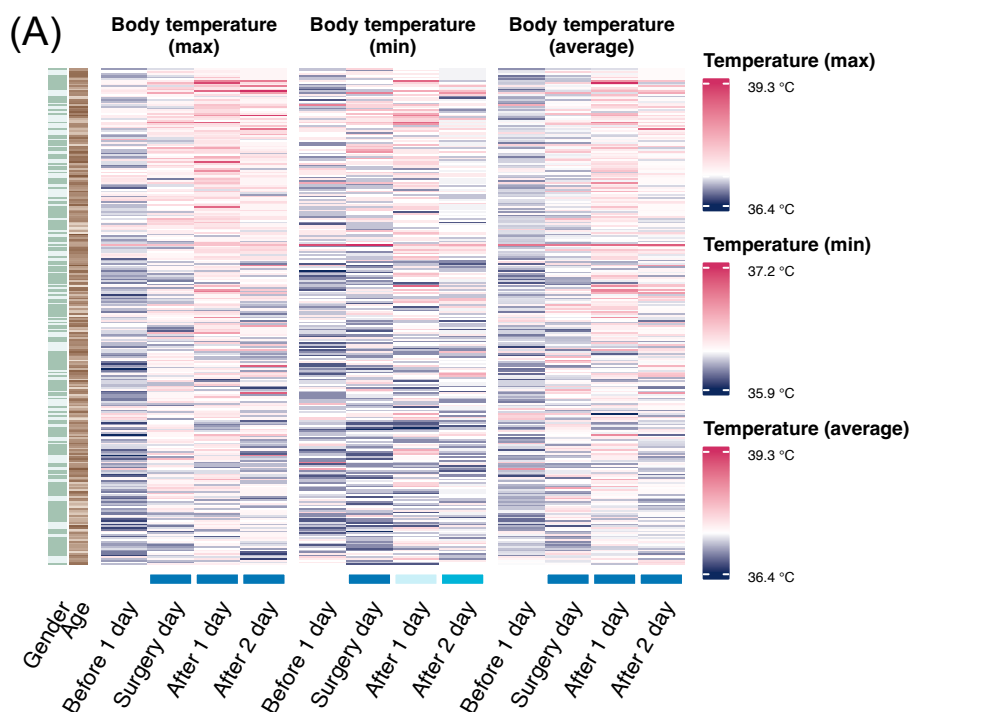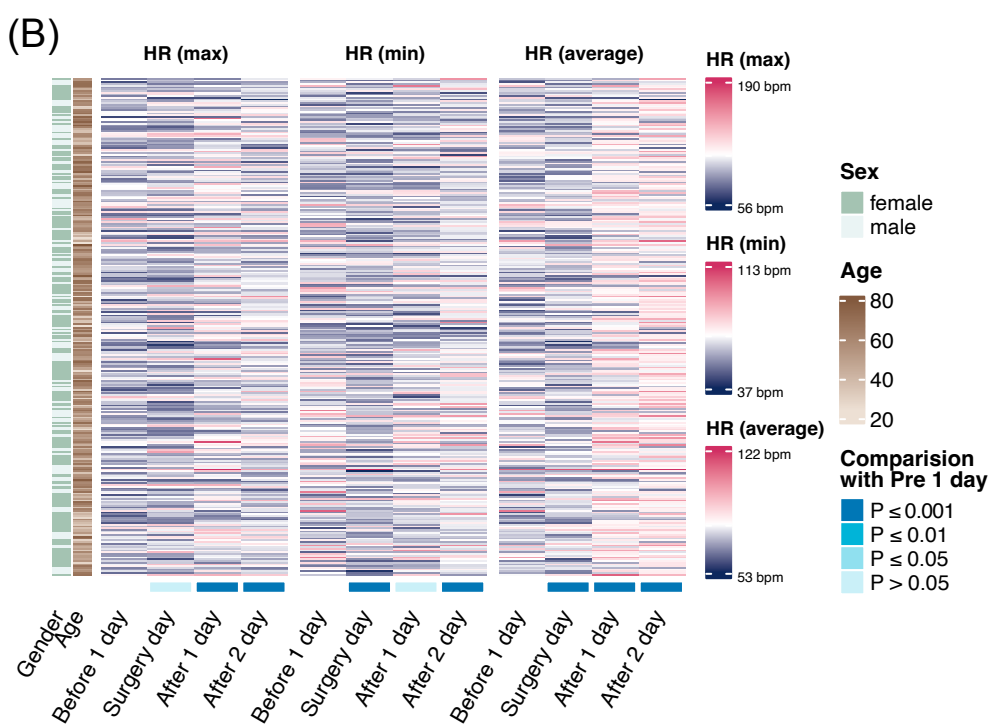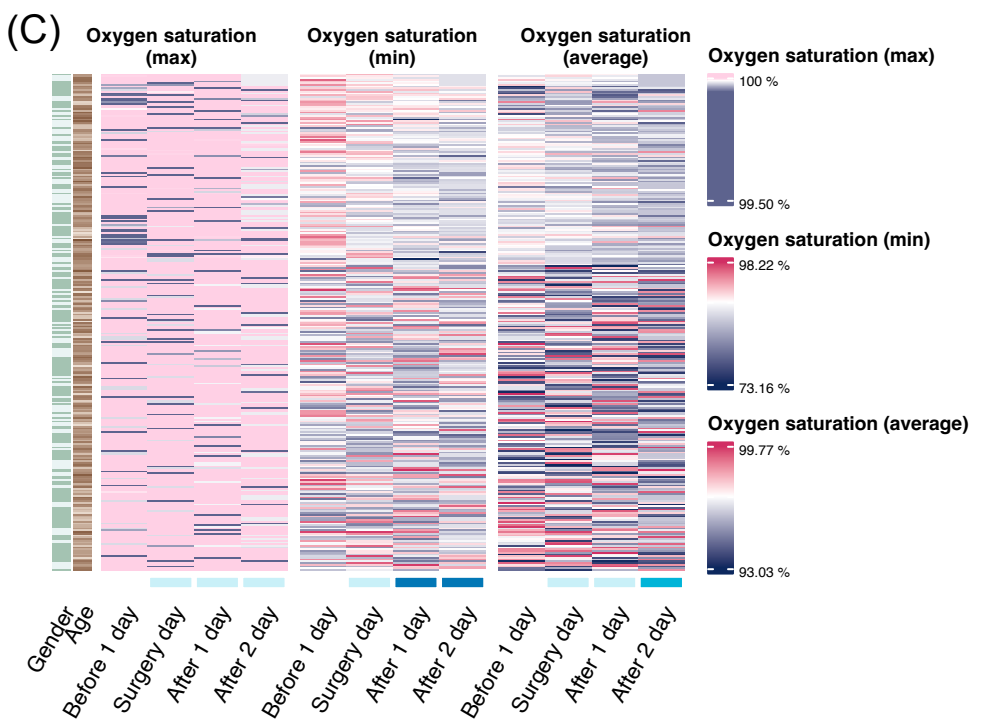

Supplement: Multimedia Appendix 1 [file mhealth_v13i1e69512_app1.zip › Vector Figures Package/Figure 3.pdf]

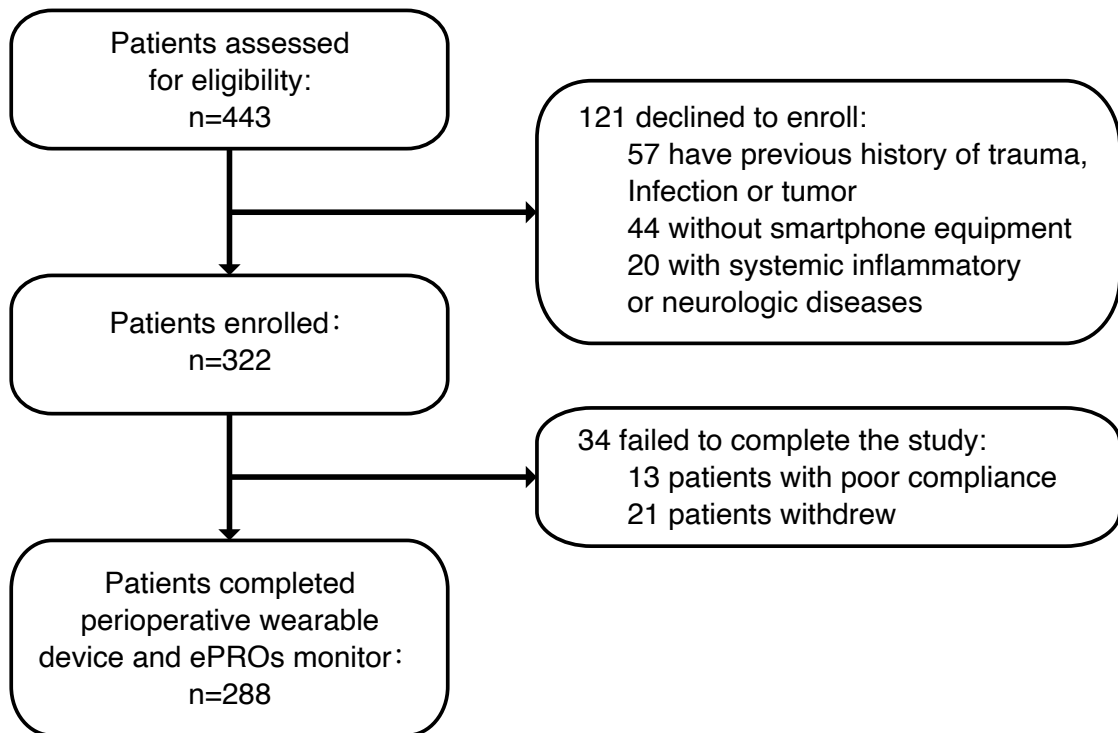

Supplement: Multimedia Appendix 1 [file mhealth_v13i1e69512_app1.zip › Vector Figures Package/Figure 2.pdf]

(A)

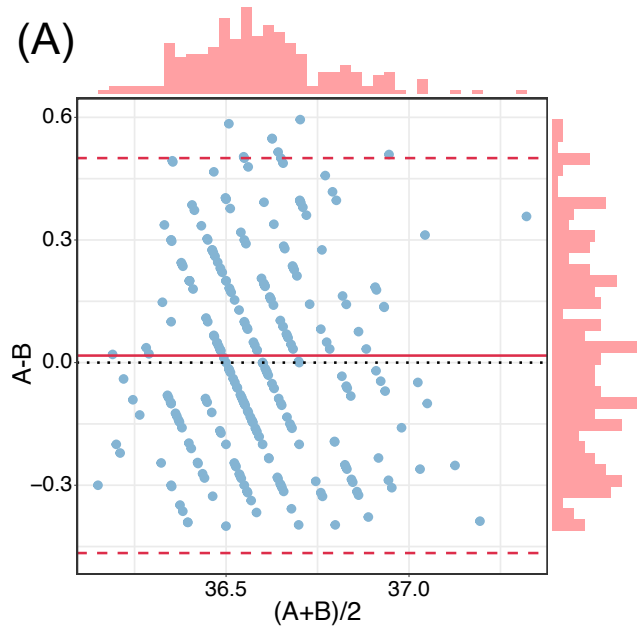

(B)

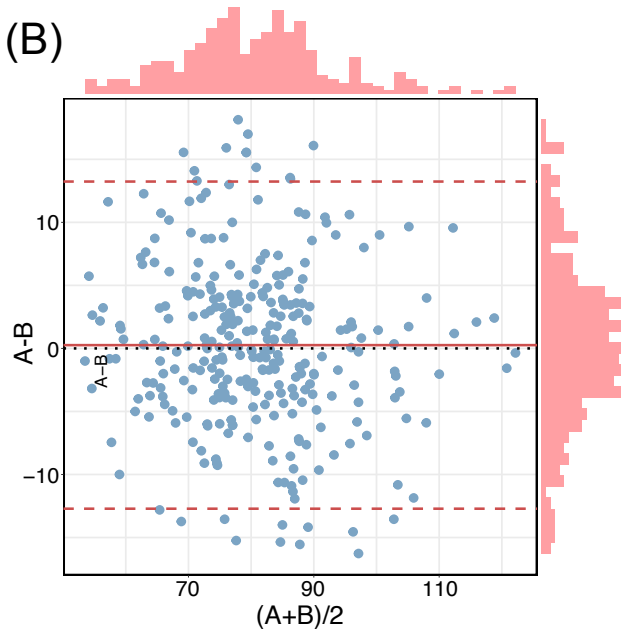

(C)

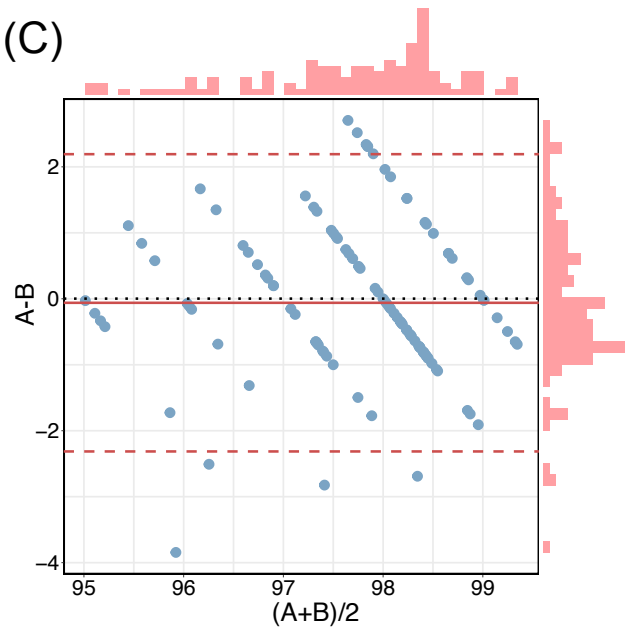

Supplement: Multimedia Appendix 1 [file mhealth_v13i1e69512_app1.zip › Vector Figures Package/Figure 5.pdf]

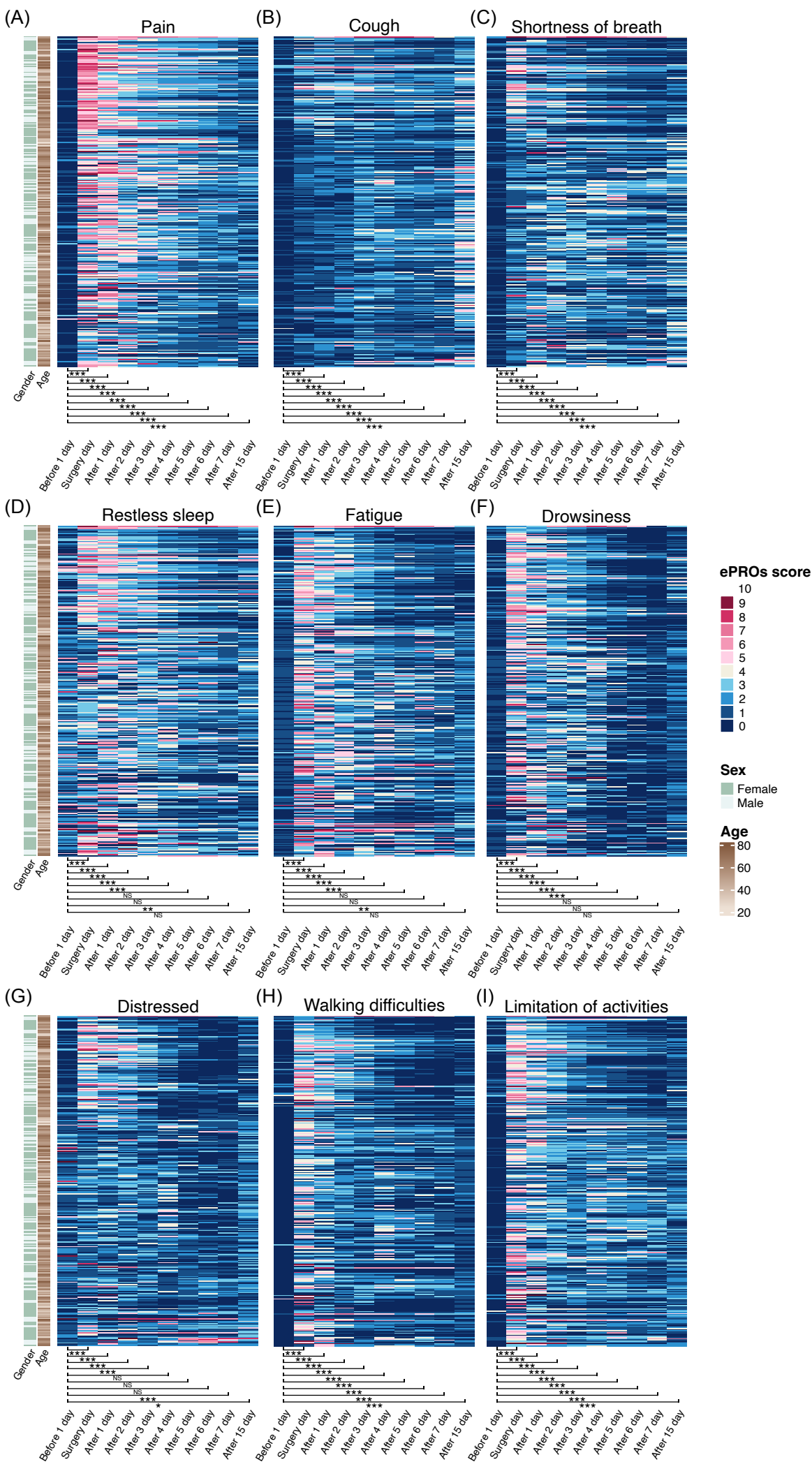

Supplement: Multimedia Appendix 1 [file mhealth_v13i1e69512_app1.zip › Vector Figures Package/Figure 4.pdf]

(A)

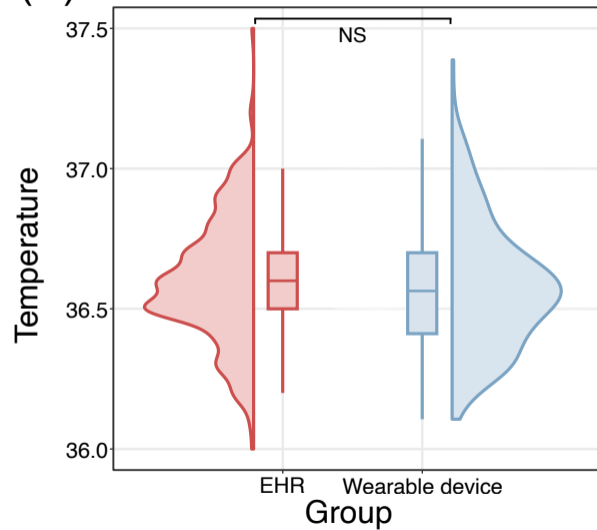

(B)

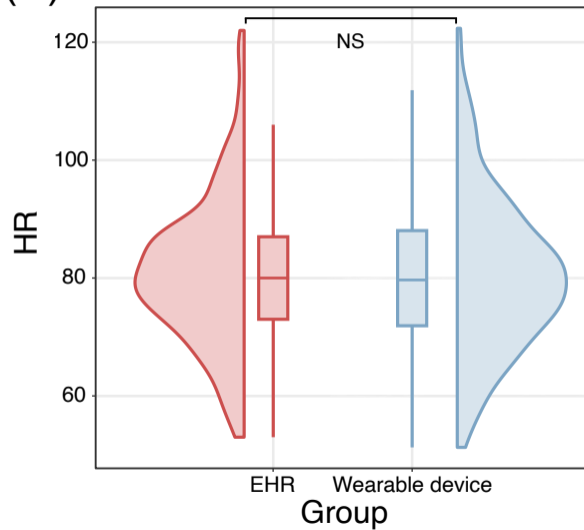

(C)

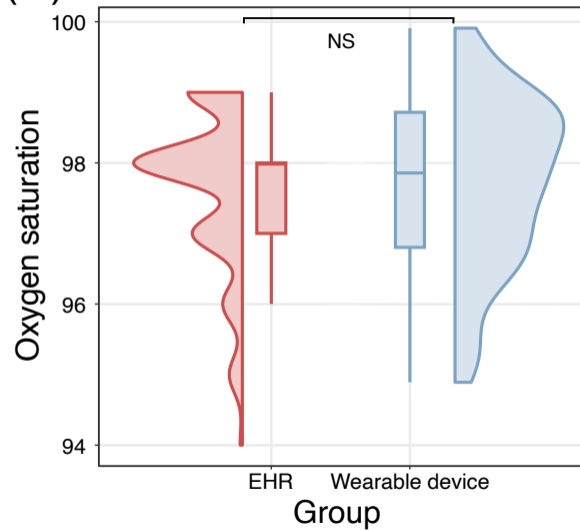

Supplement: Multimedia Appendix 1 [file mhealth_v13i1e69512_app1.zip › Vector Figures Package/Figure 6.pdf]
