# Supplementary material for: Integrating a Multimodal Digital Device for Continuous Perioperative Monitoring in Patients With Lung Cancer Undergoing Thoracic Surgery: Development and Usability Study
Source: JMIR Mhealth Uhealth. 2025 Sep 16;13:e69512. doi: 10.2196/69512 (PMC12485267; doi:10.2196/69512)
Supplement: Multimedia Appendix 3 [file mhealth_v13i1e69512_app3.docx]

Supplementary Table 1. Median and Interquartile Range Distribution of electronic patients-report outcome.

|  | Median | U-IQR | L-IQR |
| --- | --- | --- | --- |
| Pain |  |  |  |
| Pre 1 day | 0 | 0 | 0 |
| Surgery day | 5 | 3 | 7 |
| Post 1 day | 4 | 3 | 6 |
| Post 2 day | 3 | 2 | 5 |
| Post 3 day | 3 | 2 | 4 |
| Post 4 day | 2 | 1 | 3 |
| Post 5 day | 2 | 1 | 3 |
| Post 6 day | 2 | 1 | 3 |
| Post 7 day | 1 | 1 | 2 |
| Post 15 day | 1 | 0 | 2 |
| Cough |  |  |  |
| Pre 1 day | 0 | 0 | 1 |
| Surgery day | 1 | 0 | 2 |
| Post 1 day | 1 | 0 | 2 |
| Post 2 day | 1 | 0 | 2 |
| Post 3 day | 2 | 1 | 3 |
| Post 4 day | 1 | 1 | 2 |
| Post 5 day | 1 | 1 | 2 |
| Post 6 day | 1 | 1 | 2 |
| Post 7 day | 1 | 1 | 2 |
| Post 15 day | 2 | 1 | 4 |
| Shortness of breath |  |  |  |
| Pre 1 day | 0 | 0 | 1 |
| Surgery day | 2 | 1 | 3 |
| Post 1 day | 2 | 1 | 3 |
| Post 2 day | 2 | 1 | 3 |
| Post 3 day | 2 | 1 | 3 |
| Post 4 day | 1 | 1 | 3 |
| Post 5 day | 1 | 0 | 3 |
| Post 6 day | 1 | 0 | 2 |
| Post 7 day | 1 | 0 | 2 |
| Post 15 day | 2 | 1 | 3 |
| Restless sleep |  |  |  |
| Pre 1 day | 2 | 1 | 2.25 |
| Surgery day | 3 | 2 | 5 |
| Post 1 day | 3 | 2 | 5 |
| Post 2 day | 3 | 1 | 4 |
| Post 3 day | 3 | 1.75 | 4 |
| Post 4 day | 2 | 1 | 3 |
| Post 5 day | 2 | 1 | 3 |
| Post 6 day | 1 | 1 | 3 |
| Post 7 day | 1 | 0 | 2 |
| Post 15 day | 2 | 1 | 3 |
| Fatigue |  |  |  |
| Pre 1 day | 1 | 0 | 1 |
| Surgery day | 4 | 2 | 6 |
| Post 1 day | 3 | 2 | 5 |
| Post 2 day | 2 | 1 | 4 |
| Post 3 day | 2 | 1 | 3 |
| Post 4 day | 1 | 1 | 3 |
| Post 5 day | 1 | 1 | 3 |
| Post 6 day | 1 | 0 | 2 |
| Post 7 day | 1 | 0 | 2 |
| Post 15 day | 1 | 0 | 2 |
| Drowsiness |  |  |  |
| Pre 1 day | 0 | 0 | 1 |
| Surgery day | 4 | 2 | 5 |
| Post 1 day | 3 | 1 | 4 |
| Post 2 day | 2 | 1 | 3 |
| Post 3 day | 2 | 1 | 3 |
| Post 4 day | 1 | 0 | 3 |
| Post 5 day | 0 | 0 | 1 |
| Post 6 day | 0 | 0 | 1 |
| Post 7 day | 0 | 0 | 1 |
| Post 15 day | 1 | 0 | 2 |
| Distressed |  |  |  |
| Pre 1 day | 1 | 0 | 2 |
| Surgery day | 2 | 1 | 3 |
| Post 1 day | 2 | 1 | 3 |
| Post 2 day | 2 | 1 | 3 |
| Post 3 day | 1 | 1 | 2 |
| Post 4 day | 1 | 0 | 2 |
| Post 5 day | 1 | 0 | 1 |
| Post 6 day | 1 | 0 | 1 |
| Post 7 day | 0 | 0 | 1 |
| Post 15 day | 1 | 1 | 2 |
| Walking difficulties |  |  |  |
| Pre 1 day | 0 | 0 | 0 |
| Surgery day | 3 | 2 | 5 |
| Post 1 day | 2 | 1 | 4 |
| Post 2 day | 2 | 1 | 3 |
| Post 3 day | 1 | 0 | 2 |
| Post 4 day | 1 | 0 | 2 |
| Post 5 day | 1 | 0 | 2 |
| Post 6 day | 1 | 0 | 2 |
| Post 7 day | 0 | 0 | 1 |
| Post 15 day | 1 | 0 | 1 |
| Limitation of activities |  |  |  |
| Pre 1 day | 0 | 0 | 0 |
| Surgery day | 4 | 2 | 5 |
| Post 1 day | 3 | 1 | 4 |
| Post 2 day | 2 | 1 | 3 |
| Post 3 day | 2 | 1 | 3 |
| Post 4 day | 2 | 1 | 3 |
| Post 5 day | 1 | 1 | 2 |
| Post 6 day | 1 | 0 | 2 |
| Post 7 day | 1 | 0 | 2 |
| Post 15 day | 1 | 0 | 2 |

U-IQR: Upper Half of IQR; L-IQR: Lower Half of IQR.
