# Supplementary material for: Integrating a Multimodal Digital Device for Continuous Perioperative Monitoring in Patients With Lung Cancer Undergoing Thoracic Surgery: Development and Usability Study
Source: JMIR Mhealth Uhealth. 2025 Sep 16;13:e69512. doi: 10.2196/69512 (PMC12485267; doi:10.2196/69512)
Supplement: Multimedia Appendix 4 [file mhealth_v13i1e69512_app4.docx]

Supplementary Table 2. Results of threshold analysis based on the ePROs overall score.

| Character | High score  group | |  | Low score  group | |  | P-value |
| --- | --- | --- | --- | --- | --- | --- | --- |
|  | Mean | SD |  | Mean | SD |  |  |
| Body temperature (°C) |  |  |  |  |  |  |  |
| Pre 1d max | 36.97 | 0.26 |  | 37.00 | 0.27 |  | 0.43 |
| Pre 1d min | 36.24 | 0.15 |  | 36.24 | 0.17 |  | 0.75 |
| Pre 1d average | 36.58 | 0.18 |  | 36.59 | 0.23 |  | 0.50 |
| Surg max | 37.22 | 0.27 |  | 37.16 | 0.29 |  | 0.07 |
| Surg min | 36.30 | 0.17 |  | 36.22 | 0.16 |  | < 0.001 |
| Surg average | 36.72 | 0.20 |  | 36.68 | 0.22 |  | 0.11 |
| Post 1d max | 37.40 | 0.40 |  | 37.26 | 0.37 |  | < 0.01 |
| Post 1d min | 36.35 | 0.18 |  | 36.31 | 0.17 |  | 0.09 |
| Post 1d average | 36.82 | 0.24 |  | 36.79 | 0.25 |  | 0.30 |
| Post 2d max | 37.29 | 0.50 |  | 37.15 | 0.37 |  | 0.01 |
| Post 2d min | 36.29 | 0.15 |  | 36.27 | 0.13 |  | 0.24 |
| Post 2d average | 36.76 | 0.23 |  | 36.71 | 0.22 |  | 0.11 |
| Skin temperature (°C) |  |  |  |  |  |  |  |
| Pre 1d max | 34.55 | 0.70 |  | 34.50 | 0.66 |  | 0.59 |
| Pre 1d min | 31.25 | 0.83 |  | 31.31 | 0.89 |  | 0.56 |
| Pre 1d average | 33.29 | 0.65 |  | 33.21 | 0.71 |  | 0.32 |
| Surg max | 35.13 | 0.84 |  | 35.12 | 0.84 |  | 0.95 |
| Surg min | 30.93 | 1.17 |  | 30.84 | 1.20 |  | 0.54 |
| Surg average | 33.42 | 0.88 |  | 33.51 | 0.83 |  | 0.40 |
| Post 1d max | 35.70 | 0.77 |  | 35.59 | 0.85 |  | 0.24 |
| Post 1d min | 31.63 | 1.15 |  | 31.49 | 1.28 |  | 0.37 |
| Post 1d average | 34.03 | 0.80 |  | 34.00 | 0.80 |  | 0.77 |
| Post 2d max | 35.73 | 0.80 |  | 35.69 | 0.91 |  | 0.79 |
| Post 2d min | 32.16 | 1.80 |  | 32.37 | 1.93 |  | 0.41 |
| Post 2d average | 34.23 | 1.14 |  | 34.28 | 1.19 |  | 0.76 |
| HR (bpm) |  |  |  |  |  |  |  |
| Pre 1d max | 101.18 | 16.21 |  | 92.35 | 15.20 |  | < 0.001 |
| Pre 1d min | 63.30 | 11.53 |  | 67.48 | 12.28 |  | < 0.001 |
| Pre 1d average | 75.35 | 9.43 |  | 75.46 | 9.96 |  | 0.93 |
| Surg max | 104.00 | 19.39 |  | 92.00 | 16.96 |  | < 0.001 |
| Surg min | 59.78 | 9.68 |  | 63.75 | 10.09 |  | 0.001 |
| Surg average | 74.84 | 7.14 |  | 74.24 | 9.10 |  | 0.55 |
| Post 1d max | 111.12 | 19.29 |  | 102.08 | 17.77 |  | 0.43 |
| Post 1d min | 63.56 | 9.83 |  | 66.80 | 10.71 |  | 0.03 |
| Post 1d average | 82.04 | 9.06 |  | 81.46 | 9.26 |  | 0.02 |
| Post 2d max | 108.05 | 16.44 |  | 99.82 | 17.35 |  | < 0.001 |
| Post 2d min | 67.11 | 10.97 |  | 67.94 | 10.25 |  | 0.56 |
| Post 2d average | 84.78 | 9.71 |  | 82.03 | 10.63 |  | 0.04 |
| Oxygen saturation (%) |  |  |  |  |  |  |  |
| Pre 1d max | 99.85 | 0.49 |  | 99.77 | 0.64 |  | 0.31 |
| Pre 1d min | 89.99 | 4.21 |  | 88.90 | 4.63 |  | 0.04 |
| Pre 1d average | 96.69 | 1.59 |  | 96.37 | 1.65 |  | 0.11 |
| Surg max | 99.78 | 0.71 |  | 99.85 | 0.47 |  | 0.34 |
| Surg min | 89.40 | 3.75 |  | 88.15 | 4.11 |  | < 0.01 |
| Surg average | 96.56 | 1.37 |  | 96.15 | 1.75 |  | 0.03 |
| Post 1d max | 99.85 | 0.53 |  | 99.90 | 0.44 |  | 0.43 |
| Post 1d min | 87.58 | 3.88 |  | 88.72 | 4.69 |  | 0.03 |
| Post 1d average | 96.09 | 1.37 |  | 96.53 | 1.75 |  | 0.02 |
| Post 2d max | 99.85 | 0.48 |  | 99.86 | 0.57 |  | 0.88 |
| Post 2d min | 87.94 | 3.88 |  | 87.70 | 4.64 |  | 0.67 |
| Post 2d average | 96.01 | 1.52 |  | 96.11 | 1.75 |  | 0.66 |
| Sleep quality score |  |  |  |  |  |  |  |
| Pre 1d | 74.55 | 7.51 |  | 77.83 | 7.00 |  | < 0.001 |
| Surg | 78.96 | 6.10 |  | 78.43 | 6.38 |  | 0.49 |
| Post 1d | 78.12 | 5.96 |  | 77.71 | 6.40 |  | 0.60 |
| Shallow sleep duration (min) |  |  |  |  |  |  |  |
| Pre 1d | 245.54 | 73.356 |  | 246.66 | 71.820 |  | 0.900 |
| Surg | 249.57 | 67.279 |  | 237.67 | 63.379 |  | 0.143 |
| Post 1d | 246.40 | 67.214 |  | 255.32 | 58.510 |  | 0.263 |
| Deep sleep duration (min) |  |  |  |  |  |  |  |
| Pre 1d | 129.73 | 51.418 |  | 136.55 | 50.873 |  | 0.276 |
| Surg | 146.67 | 48.472 |  | 137.65 | 49.295 |  | 0.138 |
| Post 1d | 143.26 | 47.442 |  | 136.82 | 50.473 |  | 0.301 |
| Dreaming duration (min) |  |  |  |  |  |  |  |
| Pre 1d | 92.09 | 37.18 |  | 90.97 | 40.40 |  | 0.81 |
| Surg | 93.81 | 39.51 |  | 88.77 | 40.54 |  | 0.31 |
| Post 1d | 88.07 | 36.97 |  | 87.37 | 38.57 |  | 0.88 |
| Awake duration (min) |  |  |  |  |  |  |  |
| Pre 1d | 49.06 | 46.02 |  | 50.96 | 45.58 |  | 0.73 |
| Surg | 46.35 | 39.11 |  | 43.56 | 39.56 |  | 0.57 |
| Post 1d | 49.05 | 44.44 |  | 46.11 | 42.55 |  | 0.59 |
| Total sleep duration (min) |  |  |  |  |  |  |  |
| Pre 1d | 544.58 | 145.92 |  | 560.93 | 131.97 |  | 0.34 |
| Surg | 549.11 | 123.47 |  | 549.11 | 123.47 |  | 0.13 |
| Post 1d | 548.50 | 108.95 |  | 559.91 | 126.81 |  | 0.45 |
| Daytime sleep duration (min) |  |  |  |  |  |  |  |
| Pre 1d | 77.21 | 68.58 |  | 86.76 | 68.61 |  | 0.26 |
| Surg | 82.29 | 67.28 |  | 85.02 | 62.15 |  | 0.73 |
| Post 1d | 70.78 | 60.86 |  | 80.40 | 71.19 |  | 0.25 |
| Step counts (steps) |  |  |  |  |  |  |  |
| surg | 1109.89 | 1003.20 |  | 1188.59 | 1189.45 |  | 0.56 |
| Post 1d | 2667.13 | 2027.70 |  | 2732.56 | 2507.42 |  | 0.82 |
| Exercise distance (meter) |  |  |  |  |  |  |  |
| surg | 667.31 | 535.97 |  | 721.65 | 646.90 |  | 0.45 |
| Post 1d | 1595.06 | 1166.61 |  | 1598.17 | 1375.52 |  | 0.98 |
| Calories consumption (cal) |  |  |  |  |  |  |  |
| surg | 26.99 | 24.86 |  | 29.37 | 28.57 |  | 0.47 |
| Post 1d | 68.78 | 60.45 |  | 37.29 | 0.50 |  | 0.01 |

SD: standard deviation; HR: heart rate.
