# Supplementary material for: Integrating a Multimodal Digital Device for Continuous Perioperative Monitoring in Patients With Lung Cancer Undergoing Thoracic Surgery: Development and Usability Study
Source: JMIR Mhealth Uhealth. 2025 Sep 16;13:e69512. doi: 10.2196/69512 (PMC12485267; doi:10.2196/69512)
Supplement: Multimedia Appendix 6 [file mhealth_v13i1e69512_app6.docx]

Supplementary Table 4. Baseline characteristic of patients with postoperative complications.

| Characteristic | Median / Number | Range / % |
| --- | --- | --- |
| Gender |  |  |
| Female | 8 | 0.32 |
| Male | 17 | 0.68 |
| Age (years) | 60.12 | 10.92 |
| BMI |  |  |
| < 18.5 | 0 | 0 |
| 18.5 – 24.9 | 21 | 0.84 |
| ≥ 25 | 4 | 0.16 |
| Smoking history |  |  |
| Former smoker | 6 | 0.24 |
| Never smoker | 19 | 0.76 |
| Alcohol use history |  |  |
| Current alcohol use | 4 | 0.16 |
| Never drank alcohol | 21 | 0.84 |
| Preoperative comorbidities |  |  |
| Hypertension | 7 | 0.28 |
| Diabetes mellitus | 5 | 0.20 |
| COPD | 2 | 0.08 |
| Arrhythmia | 1 | 0.04 |
| CAD | 2 | 0.08 |
| Valvular heart disease | 1 | 0.04 |
| Family history of cancer |  |  |
| Yes | 9 | 0.36 |
| No | 16 | 0.64 |
| Surgical position |  |  |
| LUL | 6 | 0.24 |
| LLL | 3 | 0.12 |
| RUL | 10 | 0.40 |
| RML | 2 | 0.08 |
| RLL | 2 | 0.08 |
| LUL+LLL | 0 | 0 |
| RUL+RML | 0 | 0 |
| RUL+RLL | 2 | 0.08 |
| RML+RLL | 0 | 0 |
| RUL+RML+RLL | 0 | 0 |
| Surgical method |  |  |
| Lobectomy | 16 | 0.64 |
| Segmentectomy | 9 | 0.36 |
| Anesthesia method |  |  |
| Spontaneous ventilation | 7 | 0.28 |
| Mechanical ventilation | 18 | 0.72 |
| T stage |  |  |
| Tis | 0 | 0 |
| 1 | 11 | 0.44 |
| 2 | 8 | 0.32 |
| 3 | 5 | 0.20 |
| 4 | 1 | 0.04 |
| N stage |  |  |
| Nx | 0 | 0 |
| 0 | 15 | 0.6 |
| 1 | 4 | 0.16 |
| 2 | 5 | 0.20 |
| 3 | 1 | 0.04 |
| M stage |  |  |
| 0 | 24 | 0.96 |
| 1 | 1 | 0.04 |
| ASA grade |  |  |
| I | 0 | 0 |
| II | 18 | 0.72 |
| III | 7 | 0.28 |
| Surgical time (hours) | 2.41 | 0.92 |
| Anesthesia time (hours) | 3.70 | 1.03 |
| Intraoperative blood loss (mL) | 32.6 | 95.84 |

BMI: body mass index; CAD: coronary artery disease; COPD: Chronic obstructive pulmonary disease; LUL: left upper lobe; LLL: left lower lobe; RUL: right upper lobe; RML: right middle lobe; RLL: right lower lobe; T: tumor; N: node; M: metastasis; ASA: American society of anesthesiologists.
