# Supplementary material for: Integrating a Multimodal Digital Device for Continuous Perioperative Monitoring in Patients With Lung Cancer Undergoing Thoracic Surgery: Development and Usability Study
Source: JMIR Mhealth Uhealth. 2025 Sep 16;13:e69512. doi: 10.2196/69512 (PMC12485267; doi:10.2196/69512)
Supplement: Multimedia Appendix 9 [file mhealth_v13i1e69512_app9.docx]

Supplementary Table 5. Baseline characteristic of patients without postoperative complications.

| Characteristic | Median / Number | Range / % |
| --- | --- | --- |
| Gender |  |  |
| Female | 166 | 0.63 |
| Male | 97 | 0.37 |
| Age (years) | 55 | 24 - 82 |
| BMI |  |  |
| < 18.5 | 20 | 0.08 |
| 18.5 – 24.9 | 180 | 0.68 |
| ≥ 25 | 63 | 0.24 |
| Smoking history |  |  |
| Former smoker | 31 | 0.12 |
| Never smoker | 232 | 0.88 |
| Alcohol use history |  |  |
| Current alcohol use | 23 | 0.09 |
| Never drank alcohol | 240 | 0.91 |
| Preoperative comorbidities |  |  |
| Hypertension | 22 | 0.08 |
| Diabetes mellitus | 18 | 0.07 |
| COPD | 15 | 0.06 |
| Arrhythmia | 6 | 0.02 |
| CAD | 2 | 0.01 |
| Valvular heart disease | 2 | 0.01 |
| Family history of cancer |  |  |
| Yes | 37 | 0.14 |
| No | 226 | 0.86 |
| Surgical position |  |  |
| LUL | 64 | 0.24 |
| LLL | 30 | 0.11 |
| RUL | 63 | 0.24 |
| RML | 17 | 0.06 |
| RLL | 47 | 0.18 |
| LUL+LLL | 17 | 0.06 |
| RUL+RML | 6 | 0.02 |
| RUL+RLL | 14 | 0.05 |
| RML+RLL | 3 | 0.01 |
| RUL+RML+RLL | 2 | 0.01 |
| Surgical method |  |  |
| Lobectomy | 69 | 0.26 |
| Segmentectomy | 194 | 0.74 |
| Anesthesia method |  |  |
| Spontaneous ventilation | 109 | 0.41 |
| Mechanical ventilation | 154 | 0.59 |
| T stage |  |  |
| Tis | 50 | 0.19 |
| 1 | 187 | 0.71 |
| 2 | 24 | 0.15 |
| 3 | 2 | 0.01 |
| 4 | 0 | 0 |
| N stage |  |  |
| Nx | 14 | 0.05 |
| 0 | 239 | 0.91 |
| 1 | 7 | 0.03 |
| 2 | 3 | 0.01 |
| 3 | 0 | 0 |
| M stage |  |  |
| 0 | 261 | 0.99 |
| 1 | 2 | 0.01 |
| ASA grade |  |  |
| I | 5 | 0.02 |
| II | 223 | 0.85 |
| III | 35 | 0.13 |
| Surgical time (hours) | 1.45 | 0.40 – 7.05 |
| Anesthesia time (hours) | 2.58 | 1.35 – 8.15 |
| Intraoperative blood loss (mL) | 10.00 | 0 – 1300.00 |

BMI: body mass index; CAD: coronary artery disease; COPD: Chronic obstructive pulmonary disease; LUL: left upper lobe; LLL: left lower lobe; RUL: right upper lobe; RML: right middle lobe; RLL: right lower lobe; T: tumor; N: node; M: metastasis; ASA: American society of anesthesiologists.
