# Supplementary material for: Integrating a Multimodal Digital Device for Continuous Perioperative Monitoring in Patients With Lung Cancer Undergoing Thoracic Surgery: Development and Usability Study
Source: JMIR Mhealth Uhealth. 2025 Sep 16;13:e69512. doi: 10.2196/69512 (PMC12485267; doi:10.2196/69512)
Supplement: Multimedia Appendix 1 [file Figure 7.pdf › Vector Figures Package/Figure 7.pdf]

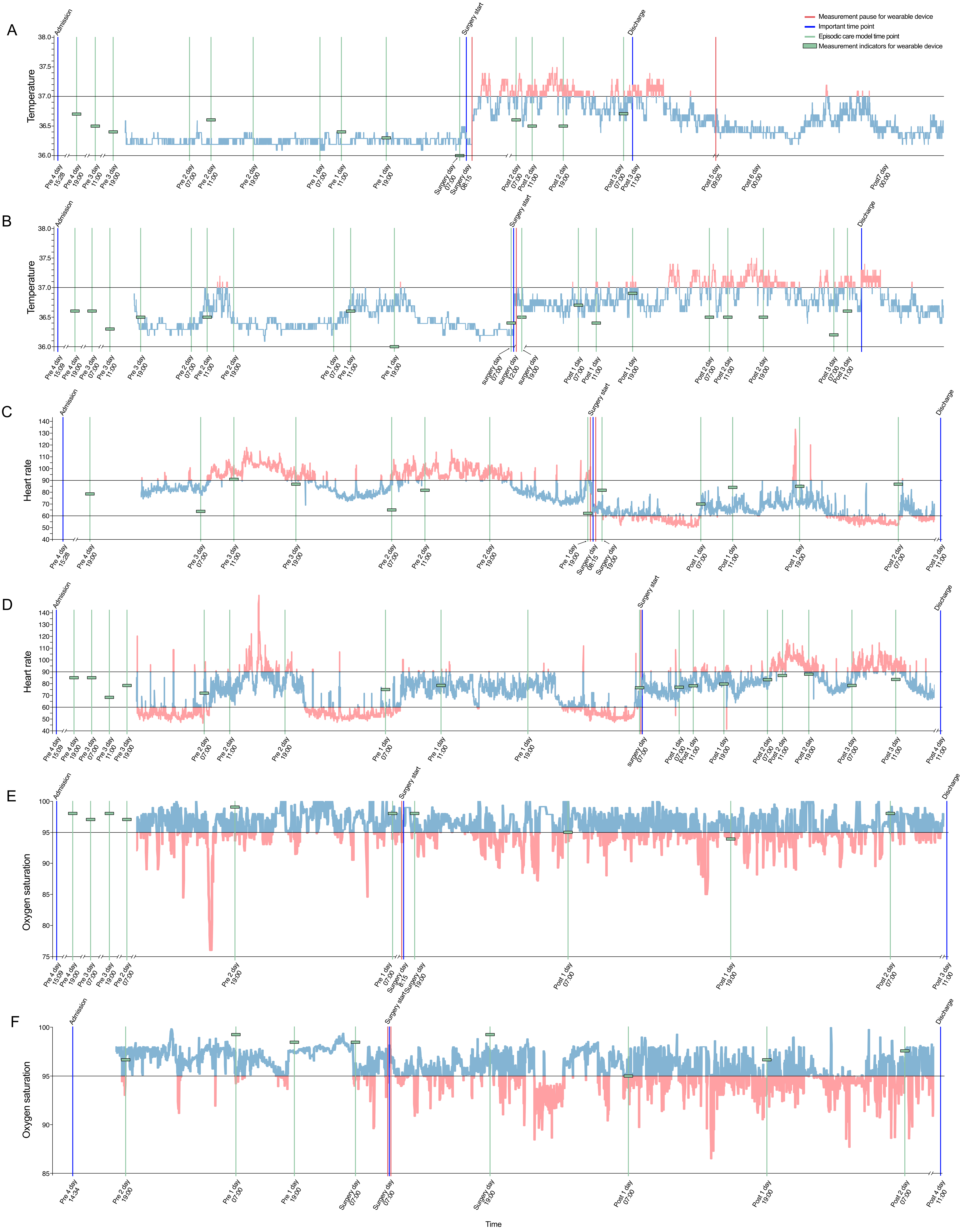
A
B
C
D
E
F
Measurement pause for wearable device
Important time point
Episodic care model time point
Measurement indicators for wearable device
Temperature
Heart rate
Oxygen saturation
Time
Admission
Surgery start
Discharge
Pre 4 day 15:28
Pre 4 day 19:00
Pre 3 day 11:00
Pre 3 day 19:00
Pre 2 day 07:00
Pre 2 day 11:00
Pre 2 day 19:00
Pre 1 day 07:00
Pre 1 day 11:00
Pre 1 day 19:00
Surgery day 07:00
Surgery day 08:15
Post 2 day 07:00
Post 2 day 11:00
Post 2 day 19:00
Post 3 day 07:00
Post 3 day 11:00
Post 5 day 09:05
Post 6 day 00:00
Post7 day 00:00
Pre 4 day 15:09
Pre 3 day 07:00
Pre 3 day 19:00
surgery day 07:00
surgery day 12:00
surgery day 19:00
Post 1 day 07:00
Post 1 day 11:00
Post 1 day 19:00
Post 2 day 07:00
Post 2 day 11:00
Post 2 day 19:00
Post 3 day 07:00
Post 3 day 11:00
Surgery day 19:00
Post 4 day 11:00
Surgery day 8:15
Pre 4 day 14:34
Surgery day 07:30
Surgery day 19:00
